# Interactions of Antibodies to the Gram-Negative Gastric Bacterium *Helicobacter pylori* with the Synaptic Calcium Sensor Synaptotagmin 5, Correlate to Impaired Vesicle Recycling in SiMa Human Neuroblastoma Cells

**DOI:** 10.1007/s12031-020-01670-0

**Published:** 2020-08-28

**Authors:** Aaron David Kleine, Bernhard Reuss

**Affiliations:** Institute for Neuroanatomy, University Medicine Göttingen Kreuzbergring 36, 37075 Göttingen, Federal Republic of Germany

**Keywords:** *Helicobacter pylori*, *Campylobacter jejuni*, Synaptotagmin 5, SiMa cells, Calcium signaling, Vesicle recycling, Tyrosine hydroxylase

## Abstract

Due to molecular mimicry, maternal antibacterial antibodies are suspected to promote neurodevelopmental changes in the offspring that finally can cause disorders like autism and schizophrenia. Using a human first trimester prenatal brain multiprotein array (MPA), we demonstrate here that antibodies to the digestive tract bacteria *Helicobacter pylori* (α-*HPy*) and *Campylobacter jejuni* (α-*CJe*) interact with different synaptic proteins, including the calcium sensor synaptotagmin 5 (Syt5). Interactions of both antisera with Syt5 were confirmed by Western blot with a HEK293-cells overexpression lysate of this protein. Immunofluorescence and Western blotting revealed SiMa cells to express Syt5, which also co-migrated with a band/spot labeled by either α-*HPy* or α-*CJe*. Functionally, a 12-h pretreatment of SiMa cells with 10 μg/ml of either α-*HPy* or α-*CJe* resulted in a significant reduction of acetylcholine(ACh)-dependent calcium signals as compared to controls. Also ACh-dependent vesicle recycling was significantly reduced in cells pretreated with either α-*HPy* or α-*CJe*. Similar effects were observed upon pretreatment of SiMa cells with Syt5-specific antibodies. In conclusion, the present study supports the view that prenatal maternal antibacterial immune responses towards *HPy* and by this to Syt5 are able to cause functional changes, which in the end might contribute also to neurodevelopmental disorders.

**Electronic supplementary material:**

The online version of this article (10.1007/s12031-020-01670-0) contains supplementary material, which is available to authorized users.

## Introduction

With a prevalence of around 0.5% in the general population (Simeone et al. 2015), schizophrenia is one of the most frequent neuropsychiatric and probably also neurodevelopmental disorders worldwide. Patients suffering from this disease are characterized by changes in cognition, emotion, and self-perception, including both negative symptoms like avolition, alogia, apathy, poor or nonexistent social functioning, and positive symptoms like hallucinations and delusions (Tandon et al. 2009). With regard to its causes, schizophrenia seem not only to depend on psychodynamic effects but also on changes in organic brain structure and functioning (Harrison 1999; Harland et al. 2009; Kahn and Sommer 2015). However, schizophrenia seems to be a multifactorial disease, and therefore, the underlying causes have still not yet been completely identified. Instead a couple of hypotheses have been formulated which still await their experimental confirmation.

One of the most fruitful of these hypotheses is the so called neurodevelopmental hypothesis for schizophrenia pathogenesis (Murray et al. 1992, 2017), which suggests the disease to start already in utero during prenatal brain development and most probably already during the late first and/or early second trimester of pregnancy (Murray et al. 2017). Based on this hypothesis on one hand, genetic factors have been investigated, leading to the identification of a large number of schizophrenia candidate genes (Hosák et al. 2012; Giusti-Rodríguez and Sullivan 2013). On the other hand, also environmental factors such as infections as well as pre- and perinatal complications during pregnancy and birth seem to play a role for schizophrenia pathology. Along this line, pre- and perinatal infections with pathogens like influenza or herpes simplex viruses, or eukaryotic endoparasites like *Toxoplasma gondii*, have already been investigated at more detail (Khandaker et al. 2013).

In the last decade, also bacterial infections, which have been suspected already during the very early days of psychiatric research to play a role in schizophrenia pathology (Noll 2004, 2007) have come anew into the focus of scientific interest (Khandaker et al. 2013; Lee et al. 2019). Thus, two population-based studies revealed that prenatal maternal infections during the first trimester of pregnancy with the Gram-negative bacterium *Neisseria gonorrhoeae* (*NGo*) correlate to an increased schizophrenia lifetime risk in the affected offspring (Babulas et al. 2006; Sørensen et al. 2009). *NGo* is widely known as a common cause for clinical and subclinical reproductive tract infections in women and men (Edwards and Butler 2011), and in these cases, antibodies directed to *NGo*-specific epitopes are a common feature of the blood serum of *NGo*-infected women (Hoffman et al. 1979). Therefore cross-reactivity of *NGo*-specific antibodies with specific brain proteins could well be responsible for perturbed brain development following exposure during early pregnancy.

A possible mechanism underlying such effects could be the so called molecular mimicry, which describes the observation that antibodies induced by an infection (Oates et al. 1977) due to molecular similarities are accidentally able to bind also to cellular proteins and by this to change or impair the functions of these proteins (Oldstone 1998). This concept has been already shown to underlie several neurological and neuropsychiatric disorders such as the Guillain-Barré syndrome that can be elicited by infections with *Campylobacter jejuni* (Speed et al. 1984; Wijdicks and Klein 2017), or Sydenham’s chorea, which is sometimes caused by infections with A-type streptococci such as *Streptococcus pyogenes* and *Streptococcus dysgalactiae* (Berrios et al. 1985; Kirvan et al. 2006; Cunningham 2014). Based on this concept, we could already previously demonstrate that polyclonal antisera directed to *NGo* are able to interact with different cellular and synaptic proteins, some of which have already been earlier identified as schizophrenia candidates (Almamy et al. 2017).

Besides the effects of gonorrhea, there are also hints available that other maternal prenatal bacterial infections could contribute to the neurodevelopmental pathology of schizophrenia (Sørensen et al. 2009; Lee et al. 2019). Thus also the microaerophilic helix-shaped Gram-negative bacterium *Helicobacter pylori* (*HPy*) has been suggested to play a role as a possible environmental factor for schizophrenia pathogenesis (Yilmaz et al. 2008). *HPy*, a Gram-positive, facultative anaerobic bacterium, is primarily found in the gastric mucosa (Montecucco and Rappuoli 2001), where it is well known as the primordial pathogenic factor for peptic ulcer disease and even gastric cancer (Caruso and Fucci 1990; Labenz and Börsch 1994). Seropositivity for *HPy* and/or the closely related bacterium *CJe* have been found to be associated with several neurological disorders, such as Parkinson’s disease (Dobbs et al. 1999), Alzheimer’s disease (Roubaud-Baudron et al. 2012; Kountouras et al. 2009), and Guillain-Barré syndrome (Kountouras et al. 2005; Moran and Prendergast 2001), whereas a negative correlation has been found for certain forms of multiple sclerosis (Li et al. 2007). Despite these neurological diseases, *HPy* has been also suspected to play a role in schizophrenia pathology (Tret'iakov et al. 2006; Yilmaz et al. 2008). This connection has been established based on the fact that *HPy* is a well-known causal agent for peptic ulcers of the stomach leading in some cases even to stomach cancer (Marshall and Warren 1983), together with the finding that schizophrenic patients frequently suffer also from ulcer disease (Tret'iakov et al. 2006) and exhibit a comparably high rate of *HPy* infections (de Hert et al. 1997).

In the present study, we intended to follow this line and started therefore to identify possible interaction partners for antibodies directed to *HPy* in the prenatal human brain on the protein level. For this, we used a commercial multiprotein array (MPA, hEXselect, Engine, Berlin, Germany) representing more than 10,000 different proteins, encoded by 23,806 expression clones of a cDNA library extracted of two late first-trimester human fetal brains (Büssow et al. 1998). By this we could identify and confirm among others synaptotagmin 5 (Syt5), a synaptic calcium sensor, and a protein with potential relevance for schizophrenia pathology (Kontkanen et al. 2002; Maycox et al. 2009; Martins-de-Souza et al. 2010) as a cellular interaction partner for α-HPy. Functional consequences of this interaction were then characterized by imaging its influence on both acetylcholine-dependent calcium transients and on synaptic vesicle recycling in SiMa neuroblastoma cells, an in vitro cell culture model for human neuronal differentiation (Marini et al. 1999).

## Materials and Methods

### Antisera and Secondary Antibodies

The following primary and secondary antibodies were used in this study: rabbit anti-*Helicobacter pylori* (**α-*HPy***; Antikoerper-online.de, cat. no. ABIN4316874); rabbit anti-*Campylobacter jejuni* (**α-*CJe***; Antikoerper-online.de, cat. no. ABIN285438); rabbit anti-*Synaptotagmin* 5 (**α-Syt5**; Antikoerper-online.de, cat. no. ABIN2422201); mouse anti-tyrosine hydroxylase (**α-TH**; Chemicon, cat. no. MAB318); mouse anti-β-actin (**α-βAct**, Sigma-Aldrich, cat. no. A2228); goat anti-rabbit IgG, peroxidase coupled (Sigma-Aldrich, cat. no. A9169); goat anti-rabbit IgG, Atto488 coupled (Sigma-Aldrich, cat. no. 18772); goat anti-mouse IgG, Atto488 coupled (Sigma-Aldrich, cat. no. 62197); and rabbit anti-mouse IgG, peroxidase coupled (Thermofisher, cat. no. 31450).

### Recombinant Proteins and Overexpression Lysates

The following cell lysates were used in this study: Untransfected **HEK293** control-lysate (Origene, cat. no. LY500001). Human **Syt5-**transfected HEK293 overexpression lysate (Origene, LY418847). Human **SLC17A7-**transfected HEK293 overexpression lysate (Origene, cat. no. LY412521). Human **STMN4**-transfected HEK293 overexpression lysate (Origene, cat. no. LY410717). Human **NCAN**-transfected HEK293 overexpression lysate (Amsbio, cat. no. LC418023). Human **SRF**-transfected HEK293 overexpression lysate (Origene, cat. no. LY418874).

### hEXselect Multiprotein Array Analysis

A high-density multiprotein array (MPA) (hEXselect, Engine, Berlin, Germany, Order No. 1003) derived from a cDNA bank of two first trimester human fetal brain samples, containing 23,806 *E. coli* expression clones representing a total of 10,000 human proteins (Büssow et al. 1998; Horn et al. 2006), was incubated with either α-*HPy* or α-*CJe* according to the manufacturer’s protocol. In brief, prior to the first use of these arrays, protein spots were fixed on the PVDF membrane for 10 min with 70% ethanol and rinsed twice with distilled water. After removing excess protein material with tissue paper and Tris-buffered saline (TBS) supplemented with 0.05% Tween 20 and 0.5% Triton X-100 (TBS-TT), the MPA was washed three times for 10 min with TBS-TT and rinsed twice for 10 min with TBS alone, followed by a 2-h blocking step with 3% skim milk powder in TBS. The MPA was then incubated overnight with the respective primary antiserum at a dilution of 1:2000 at 4 °C on a rocking shaker. On the following day, the MPA was washed three times for 10 min in TBS, containing 0.05% Tween-20 (TBS-T), before incubating it with appropriate secondary antibodies at a dilution of 1:10 000 for 2 h at room temperature. After four washes with TBS-T and two washes with TBS, the location of immunoreactive spots was visualized by exposing the membrane to a SuperRX medical X-ray film (Fuji, Düsseldorf, Germany), during the application of a chemiluminescent peroxidase substrate (0.1-mol/l Tris-HCl (pH 8.6), 0.25-mg/ml luminol, 0.2-mg/ml p-hydroxycoumaric acid, and 0.1% H_2_O_2_) in a dark room cabinet. For reprobing, the MPA membrane was stripped with 1-mol/l NaOH for 40 min and then processed for a second round of immunodetection as described. All experiments were repeated at least in duplicate. As a control experiment, the hEXselect multiprotein was incubated with secondary antibodies only, to demonstrate the antibody-specificity of the signals obtained with bacteria-specific antisera (see also Supplementary Fig. 1).

### Cell Culture

SiMa cells (human neuroblastoma; DSMZ, Braunschweig, Germany; Marini et al. 1999) were maintained in RPMI medium supplemented with 10% FCS, glutamine, and penicillin/streptomycin, in an incubator at 37 °C and under a humidified atmosphere containing 5% CO_2_. Medium was exchanged every other day, and shortly before reaching confluency, cells were mechanically suspended and seeded at a lower density either on 12-mm glass cover slips (Menzel, Braunschweig, Germany) in a 24-well plastic multiwell plate (Sarstedt, Nümbrecht, Germany) for immunocytochemistry or on 6-well plastic multiwell plates (Sarstedt, Nümbrecht, Germany) for Western blot analysis. For antibody treatment, upon reaching the requested density, cells were pre-incubated for 4 h in an RPMI medium containing 1% FCS and subsequently for a time span as indicated in the text with 10 μg/ml of the respective antiserum. Prior to use, sodium azide of the applied antisera was removed by microdialysis with Amicon Ultra filter units (Merck, Darmstadt, Germany).

### Immunocytochemistry

Immunocytochemistry was performed as described earlier (Dahm et al. 2010). In brief, cells on glass cover slips (Menzel, Braunschweig, Germany) were washed with PBS and fixed for 10′ with 4% paraformaldehyde (PFA) in PBS. After washing, cells were permeabilized for 10′ with a mixture of acetone/methanol (1:1) at − 20 °C. Following three washes with phosphate buffered saline (PBS), cells were blocked for 1 h with goat serum (GS) diluted 1:50 in PBS (PBS-GS). Primary antibodies diluted 1:50 in PBS-GS were applied overnight at 4 °C, followed by three washes with PBS and a 90′ incubation with Atto488-coupled secondary antibodies (Sigma-Aldrich, Steinheim, Germany), diluted 1:400 in PBS-GS at 37 °C. After three washes with PBS, cells were mounted on standard microscope slides using a commercial mounting medium (DAKO, Glostrup, Denmark). Imaging was performed using an Axiocam digital camera system, mounted on an Axiophot microscope (both Zeiss, Jena, Germany).

### Western Blot Analysis

Western blot analysis was performed as described previously (Dahm et al. 2010), with either 5 μg of total cellular protein, 500 ng of a recombinant protein sample, or 1 μg of an overexpression lysate being electrophoretically size separated on an 8.5% SDS polyacrylamide gel (Laemmli 1970) using a Mini Protean Gel System (Biorad, München, Germany). After tank-blot Western transfer onto a polyvinyl difluoride membrane (PVDF; Roth, Karlsruhe, Germany), blocking occurred for 1 h at 4 °C in 3% (w/v) nonfat dry milk in TBS with 0.01% Tween-20 (TBST). Blots were then incubated overnight at 4 °C with primary antibodies (as listed above), diluted 1:2000 in TBST with 0.1% nonfat dry milk. After washing, rabbit-specific peroxidase-coupled secondary antibodies (Sigma-Aldrich, Steinheim, Germany), diluted at 1:10000 in TBST, with 0.1% nonfat dry milk, were applied for 90′ at room temperature. Subsequent visualization occurred by exposing SuperRX medical X-ray films (Fuji, Düsseldorf, Germany), during the application of a peroxidase chemiluminescence substrate (0.1-mol/l Tris-HCl (pH 8.6), 0.25-mg/ml luminol, 0.2-mg/ml p-hydroxycoumaric acid, and 0.1% H_2_O_2_) to the blots. For reprobing, blots were stripped with 1-mol/l NaOH for 15′ and then processed for a second round of immunodetection as described. Each blot was repeated at least three times. Also in this case specificity controls were performed incubating similar Western blots with secondary antibodies only. By this it is becoming clear that the signals obtained with the bacteria-specific antisera are not only due to an unspecific interaction of the secondary antibodies (see also Supplementary Figs. 2, 3, and 4).

### Two-Dimensional Western Blot Analysis

Isoelectric focusing and 2D-Gel electrophoresis were performed according to a previously described method (Bollag and Edelstein 1994, see also Reuss and Asif 2014) using a Mini-Protean Gel System (Biorad, München, Germany). In brief, after cultivation on a 6-well plastic multiwell plate (Sarstedt, Nümbrecht, Germany) and before reaching confluency, cells were harvested in 2× sample collecting buffer (8-mol/l urea, 2% Triton X-100, 1% 2-mercaptoethanol, 0.1% bromophenol blue), containing also 2.4% of a commercial ampholyte solution (pH 3.5–10; Sigma-Aldrich, Steinheim, Germany). Protein concentration of the homogenate was determined densitometrically (Henkel and Bieger 1994), and for isoelectric focusing, 30 μg of total cellular protein were loaded on 5% polyacrylamide gel slices with 2.4% ampholytes (pH 3.5–10) containing also 50% urea for protein denaturation. Gel slices were then run for 30 min at 150 V followed by 2.5 h at 200 V. After equilibrating the slices for 30 min in electrophoresis sample buffer, they were located on top of an 8% SDS polyacrylamide gel followed by electrophoretic separation of the proteins according to their molecular weight. After this secondary run, Western transfer onto PVDF-membrane was performed by tank blot, and the obtained membrane was immunostained with the respective antiserum at a dilution of 1:2000. After photographic documentation, blots were stripped with 1-mol/l NaOH for 15′ and then processed for a second round of immunodetection as described. Each experiment was repeated at least three times. Also for the two-dimensional Western blot analysis, a control with secondary antibodies only reveals that the signals obtained with the bacteria-specific antisera are not detected only due to an unspecific interaction of the secondary antibodies (see also Supplementary Figs. 3 and 4).

### Calcium Imaging

For dye loading, cell cultures on 12-mm glass cover slips, with or without a 12-h pretreatment with different antibacterial antisera, were incubated for 30 min at room temperature with 5 mmol/l of the Ca^++^-sensitive fluorescent dye Fluo3-AM (Sigma) diluted in a standard bath solution (NaCl 150 mmol/l, KCl 5.4 mmol/l, CaCl_2_ 2 mmol/l, MgCl_2_ 1 mmol/l, Hepes, 10 mmol/l, glucose 10 mmol/l), the pH of which was adjusted to 7.3 with NaOH. For calcium imaging, dye-loaded cells were transferred to a recording chamber, continuously superfused with the standard bath solution at a rate of 5 ml/min. Acetylcholine (ACh) was applied as indicated in the text by changing the perfusate. Experiments were performed at room temperature (20 °C). Fluorescence imaging was performed with a CCD camera system (Princeton Instruments, Trenton, NJ) mounted on an inverted microscope equipped with epifluorescence (Axiovert, Zeiss). In order to detect Ca^++^-transients, light with a wavelength of 485 nm was used for excitation, and fluorescence was measured at an emission wavelength of 540 nm selected with a 30-nm bandpass filter. Images were acquired every 5 s and were subsequently processed with the MetaFluor image analysis software (Universal Imaging, West Chester, PA). To identify and quantify neurotransmitter-reactive cells, fluorescence intensity during stimulation (F) was compared to the fluorescence intensity immediately before stimulation (F_0_), with the ratio of both values (F/F_0_) indicating the relative increase. To cover all Ca^++^-transients during a single neurotransmitter application, maximum plots of the complete sequence of pictures taken during every application period were generated using the MetaMorph Image analysis software (Universal Imaging, West Chester, PA).

### Assay for Vesicle Turnover

Effects of α-*HPy* and α-*CJe* on acetylcholine(ACh)-elicited exocytosis and subsequent vesicle recycling by endocytosis were visualized by incubation with the fluorescent dye FM1-43 (Chowdhury et al. 2005; Gaffield and Betz 2006). Cells with or without a 12-h pretreatment with 10 μg/ml of a given antibacterial antiserum were transferred to a recording chamber, continuously superfused with a standard bath solution (at a rate of 5 ml/min). Subsequently the perfusate was changed and the cells perfused for 50 s in standard bath solution containing 10 μmol/l of FM1-43 (Sigma-Aldrich, Steinheim, Germany) first in the absence and then in the presence of 1-nmol/l ACh (Sigma-Aldrich, Steinheim, Germany). After each FM1-43 exposure, excess dye was washed away by another 50 s perfusion with standard bath solution alone, and then FM1-43 fluorescence intensity (as a measure for the amount of exocytosis and subsequent vesicle recycling) was documented photographically with a CCD camera system (Princeton Instruments, Trenton, NJ) mounted on an inverted microscope equipped with epifluorescence (Axiovert, Zeiss). ACh-dependent exocytosis and vesicle recycling activity of the cells was calculated by determining the ratio between the intensities of FM1-43 fluorescence in the presence of ACh to the FM1-43 fluorescence in the absence of this neurotransmitter.

### MTT-Assay for Cell Viability

To exclude possible effects of α-HPy and α-CJe on cell viability, a so called MTT-assay was performed (Levitz and Diamond 1985). For this, to SiMa cells pretreated with either of both antisera or to untreated controls, MTT (3-(4,5-Dimethyl-2-thiazolyl)-2,5-diphenyl-2H-tetrazolium bromide, Merck, Darmstadt, Germany) was added at a final concentration of 50 μg/ml, followed by a 2-h incubation at 37 °C under a humidified atmosphere with 5% CO_2_. After this, brightfield photographs of the stained cells were taken with an Axiocam digital camera system, mounted on an Axiophot microscope (Zeiss, Jena, Germany). Photographs were then evaluated densitometrically with the FiJi clone of the open source image analysis program ImageJ (see https://imagej.net), followed by statistical analysis of the obtained results with the free OpenOffice Calc software (see https://www.openoffice.de).

### Gene-Specific Knockdown of Syt5 in SiMa Neuroblastoma Cells

In order to confirm interactions of the antibacterial antibodies used here with Syt5, a gene-specific knockdown of Syt5 in SiMa neuroblastoma cells was performed by transfecting them with a commercial Syt5 shRNA expression vector (MISSION® pLKO.1-puro:Syt5; Merck, Darmstadt, Germany). Upon transfection this vector expresses a short sequence of the Syt5 mRNA together with a hairpin structure (5′ CCG GCC AGA GTT ACA TAG ACA AGG TCT CGA GAC CTT GTC TAT GTA ACT CTG GTT TTT 3′) leading to a selective degradation of the endogenous Syt5 mRNA. In brief, a single-cell suspension of SiMa cells was washed with serum-free RPMI without antibiotics, and seeded in 0.8-ml of the same medium on a 6-well plate (Sarstedt, Nümbrecht, Germany) at a density of 3 × 10^6^ cells per well. Five μg of plasmid DNA were diluted in 100 μl of RPMI medium and then mixed with 100 μl of a suspension of 10% Lipofectamine 2000 (Thermo Fisher Scientific, Waltham, USA) in RPMI. This suspension was then given to the cells which were then incubated overnight at 37 °C in a CO_2_-incubator. At the following day, 4 ml of RPMI medium (supplemented with FCS, glutamine, and penicillin/streptomycin, as stated above) was added, and the cells were again incubated for 24 h at 37 °C in a CO_2_-incubator. Cells were then harvested for Western blot analysis as described above. Ten (10) μg of total cellular protein was then analyzed by this latter method to clarify whether expression of Syt5 as detected by Syt5-specific antibodies, and of the Syt5-specific bands stained by either α-HPy or α-CJe were diminished in Syt5-shRNA-transfected cells. For control purposes, both cells incubated with Lipofectamine only and cells transfected with 5 μg of a non-reacting control sh-RNA vector (MISSION® pLKO.1-puro:non-mammalian-shRNA; Merck, Darmstadt, Germany) were analyzed. As a control for equal protein loading, blots were also incubated with an antibody directed to the cellular housekeeping protein β-actin.

## Results

**Fig. 1:**
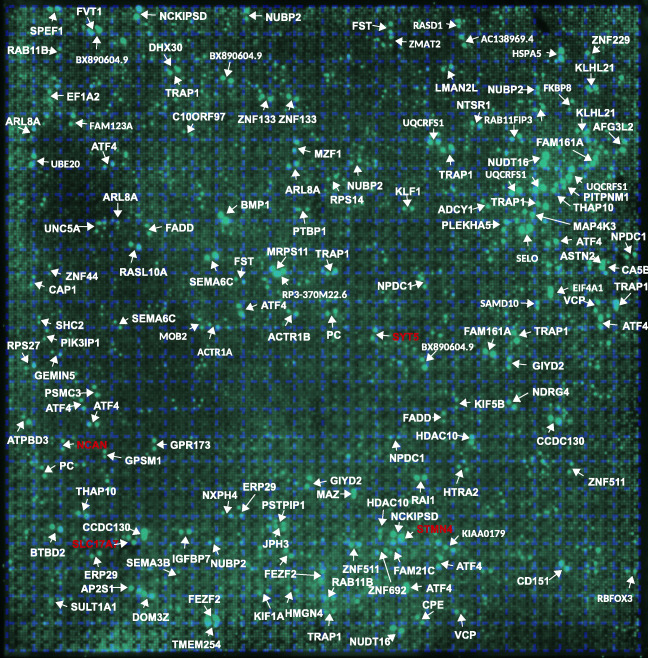
Interactions of polyclonal antibodies directed to the Gram negative microaerophilic gastric bacterium *Helicobacter pylori* (α-*HPy*) with specific protein spots on the hEXselect multiprotein array (MPA). This array contains 23,806 spots of both full-length, and shorter protein fragments, expressed in an *E. coli* system, representing a total number of around 10 000 different human proteins (Büssow et al. 2000). As revealed by the green stained false color image of an X-ray film exposed to the immune incubation of a hEXselect MPA as visualized by ECL-detection, a number of pairwise immunoreactive spots revealed immunoreactivity with α-*HPy*, which subsequently could be identified according to their membrane location as 99 different proteins (see Table 1 for a more detailed gene description). For a functional classification see also Table 3. A corresponding negative control incubated with secondary antibodies only is shown in Supplementary Fig. 1

**Fig. 2:**
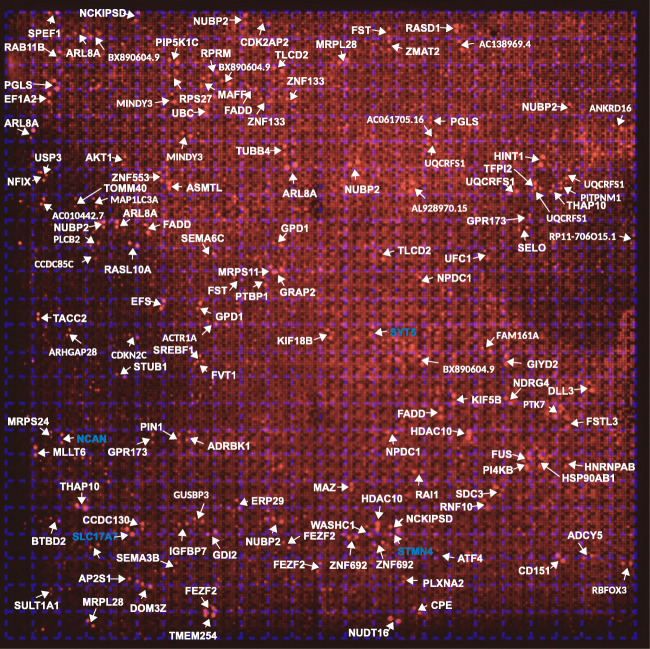
Interactions of polyclonal antibodies directed to the Gram negative gut bacterium *Campylobacter jejuni* (α-*CJe*) with specific protein spots on the hEXselect multiprotein array (MPA). This array contains 23,806 spots of both full-length, and shorter protein fragments, expressed in an *E. coli* system, representing a total number of around 10 000 different human proteins (Büssow et al. 2000). As revealed by the red stained false color image of an X-ray film exposed to the immune incubation of a hEXselect MPA as visualized by ECL-detection, a number of pairwise immunoreactive spots revealed immunoreactivity with α-*CJe*, which subsequently could be identified according to their membrane location as 107 different proteins (see Table 2 for a more detailed gene description). For a functional classification see also Table 3. A corresponding negative control incubated with secondary antibodies only is shown in Supplementary Fig. 1

In a first round of experiments, we tried to identify possible interaction partners for a polyclonal antiserum directed to the Gram-negative bacterium *Helicobacter pylori* (α-*HPy*), or the closely related bacterium *Campylobacter jejuni* (α-*CJe*) in the first trimester human fetal brain. For this we incubated a commercial hexSelect multiprotein array (MPA) covering the protein expression at this early stage of human brain development, with either α-*HPy* or α-*CJe*, and assigned the obtained immunoreactive spots to specific proteins according to the manufacturer’s instructions. For α-*HPy* (Fig. 1), a comparably high number of different immunoreactive protein spots were detected that, according to their specific location, were identified as a set of 99 different proteins (for a detailed description see Table 1) which could be further assigned to different functional groups (see also Table 3). For α-*CJe* (Fig. 2), a slightly higher number of 107 different proteins (for a detailed description see Table 2) was identified which could also be assigned to several functionally different groups (see Table 3). Of a total number of 153 identified interaction partners, 53 proteins interacted with both antisera (α-*HPy* and α-*CJe*), whereas 46 of them reacted only with α-*HPy* and 54 only with α-*CJe*. For both α-*HPy* and α-*CJe*, several established schizophrenia candidate proteins with cross-reactivity to either of these antibodies could be identified including Syt5, Slc17a7, Stmn4, and Ncan. As a control for the antibody-specificity of the signals obtained by the bacteria-specific antisera, a hEXselect multiprotein array incubated with secondary antibodies only revealed only a slight background staining (see Supplementary Fig. 1).

**Table 1:** Proteins interacting with an antiserum directed to *Helicobacter pylori*

Gene symbol	Protein name	Protein functions
AC138969.4	NPIP-like protein 1	Nuclear pore complex interacting
ACTR1A	Alpha-centractin	Microtubule-based vesicle motility
ACTR1B	Beta-centractin	Actin interacting
ADCY1	Adenylate cyclase type 1	Intracellular signaling by cAMP
AFG3L2	Paraplegin-like protein	Protease essential for axonal growth
AP2S1	AP-2 complex subunit sigma-1	Clathrin dependent endocytosis
ARL8A	ADP-ribosylation factor-like protein 8A	Axonal transport
ASTN2	Astrotactin-2	Neuron migration
ATF4	Activating transcription factor 4	Transcription factor
ATPBD3	ATP-binding domain-containing protein 3	t-RNA modification
BMP1	Bone morphogenetic protein 1	Brain development
BTBD2	BTB/POZ domain-containing protein 2	neurogenesis
BX890604.9	Uncharacterized protein ENSP00000370496 Fragment	n.a.
CA5B	Carbonic anhydrase 5B, mitochondrial	pH-regulation, carbon dioxide transport
CAP1	Adenylyl cyclase-associated protein 1	Cytoskeleton organization
CCDC130	Coiled-coil domain-containing protein 130	Spliceosome
CD151	Tspan24	Endocytosis
CPE	Carboxypeptidase E Precursor	Neuropeptide synthesis
DHX30	Putative ATP-dependent RNA helicase DHX30	Mitochondrial helicase, neurogenesis
DOM3Z	Dom-3 homolog Z	RNA-modification, decapping
EF1A2	Elongation factor 1-alpha 2	Translation regulation
EIF4A1	Eukaryotic initiation factor 4A-I	Translation
ERP29	Endoplasmic reticulum protein ERp29 Precursor	n.a.
FADD	FAS-associated death domain protein	Apoptosis signaling pathway
FAM123A	APC membrane recruitment protein 2	Ectoderm development, Wnt-signaling
FAM161A	Protein FAM161A	Microtubular organization, cilium formation
FAM21C	WASH complex subunit 2C	Intracellular vesicle transport
FEZF2	Fez family zinc finger protein 2	Dendritic arborization and spine formation
FKBP8	FK506-binding protein 8	Neural tube patterning
FST	Follistatin	Cell differentiation, embryonic development
FVT1	Follicular lymphoma variant translocation 1	Sphingolipid synthesis
GEMIN5	Gem-associated protein 5	Translation
GIYD2	Structure-specific endonuclease subunit SLX1	DNA-repair
GPR173	G protein coupled receptor 173	Neuron migration, gonadotropin signaling
GPSM1	G protein-signaling modulator 1	Nervous system development
HDAC10	Polyamine deacetylase HDAC10	Histone deacetylase activity
HMGN4	High mobility group nucleosome protein 4	Nucleosomal DNA-binding
HSPA5	Heat shock protein family A member 5	Chaperone function in the ER
HTRA2	Serine protease HTRA2, mitochondrial	Serin-type endopeptidase
IGFBP7	Insulin-like growth factor-binding protein 7	Modulation of growth factor actions
JPH3	Junctophilin-3	Synaptic plasticity
KIAA0179	Ribosomal RNA processing protein 1 homolog B	Regulation of apoptosis and transcription
KIF1A	Kinesin-like protein KIF1A	Anterograde axonal vesicle transport
KIF5B	Kinesin-like protein KIF5B	Microtubular motor protein
KLF1	Krueppel-like factor 1	Erythrocyte development
KLHL21	Kelch-like protein 21	Protein ubiquitination
LMAN2L	VIP36-like protein	Vesicle transport in the ER
MAP4K3	Mitogen-activated protein kinase kinase kinase kinase 3	MAP-kinase signaling, stress response
MAZ	Myc-associated zinc finger protein	Transcription factor-binding
MOB2	MOB kinase activator 2	Actin, Neuronal process formation
MRPS11	28S ribosomal protein S11, mitochondrial	Mitochondrial translation
MZF1	Myeloid zinc finger 1	Transcription factor
NCAN*	Neurocan	Extracellular matrix, neural development
NCKIPSD	NCK-interacting protein with SH3 domain	Actin cytoskeleton, Neurite formation
NDRG4	N-myc downstream-regulated gene 4 protein	Brain development
NPDC1	Neural proliferation differentiation and control protein 1	Neural stem cell proliferation
NTSR1	Neurotensin receptor type 1	Peptidergic neurotransmission
NUBP2	Nucleotide-binding protein 2	Neurite formation
NUDT16	U8 snoRNA-decapping enzyme	RNA-decapping enzyme
NXPH4	Neurexophilin-4	Neuropeptide signaling
PC	Pyruvate carboxylase, mitochondrial	Gluconeogenesis
PIK3IP1	Phosphoinositide-3-kinase-interacting protein 1	Intracellular signaling
PITPNM1	Membrane-assoc. phosphatidylinositol transfer protein 1	Cytoskeleton, brain development
PLEKHA5	Pleckstrin homology domain-cont. Family A member 5	Intracellular signaling
PSMC3	26S proteasome regulatory subunit 6A	Protein degradation
PSTPIP1	Prol-ser-threo phosphatase-interacting protein 1	Actin cytoskeleton remodeling
PTBP1	Polypyrimidine tract-binding protein 1	pre-mRNA-splicing
RAB11B	Ras-related protein Rab-11B	Intracellular vesicle trafficking, autoimmunity
RAB11FIP3	Rab11 family-interacting protein 3	Intracellular vesicle trafficking, cytokinesis
RAI1	Retinoic acid-induced protein 1	Transcription, Circadianic clock, Neurodvelopm.
RASD1	Dexamethasone-induced Ras-related protein 1	Transcription factor
RASL10A	Ras-like protein family member 10A	G Protein, intracellular signaling
RBFOX3	RNA binding protein fox-1 homolog 3	Splicing regulator, neuronal development
RP3-370 M22.6	N.N.	n.a.
RPS14	40S ribosomal protein S14	Translation
RPS27	40S ribosomal protein S27	Translation
SAMD10	Sterile alpha motif domain-containing protein 10	Nuclear localization
SELO	Selenoprotein O	Protein adenylation
SEMA3B	Semaphorin-3B	Axon guidance
SEMA6C	Semaphorin-6C	Axon guidance
SHC2	SH2 domain-containing-transforming protein C2	MAP-kinase signaling
SLC17A7*	Vesicular glutamate transporter 1	Presynaptic glutamate reuptake
SPEF1	Sperm flagellar protein 1	Cell migration
STMN4*	Stathmin-4	Microtubular, neurite formation
SULT1A1	Sulfotransferase 1A1	Catecholamine metabolism
SYT5*	Synaptotagmin 5	Synaptic vesicle recycling
THAP10	THAP domain-containing protein 10	Transcription factor
TMEM254	Transmembrane protein 254	n.a.
TRAP1	Heat shock protein 75 kDa, mitochondrial	Mitochondrial chaperone, cell respiration
UBE20	(E3-independent) E2 ubiquitin-conjugating enzyme	Ubiquitinylation
UNC5A	Netrin receptor UNC5A	Netrin signaling, neurite outgrowth
UQCRFS1	Cytochrome b-c1 complex subunit Rieske, mitochondrial	Respiratory chain functioning
VCP	Valosin-containing protein	Protein quality control
ZMAT2	Zinc finger matrin-type protein 2	mRNA splicing
ZNF133	Zinc finger protein 133	Transcription factor
ZNF229	Zinc finger protein 229	Transcription factor
ZNF44	Zinc finger protein 44	Transcription factor
ZNF511	Zinc finger protein 511	Transcription factor
ZNF692	Zinc finger protein 692	Transcription factor

Known schizophrenia candidates are labeled by an asterisk (*)

**Table 2:** Proteins interacting with an antiserum directed to *Campylobacter jejuni*

Gene symbol	Protein name	Protein functions
AC010442.7	Putative uncharacterized protein LOC116349 Precursor	n.a.
AC061705.16	Uncharacterized protein ENSP00000380804	n.a.
AC138969.4	NPIP-like protein 1	Nuclear pore complex interacting
ACTR1A	Alpha-centractin	Microtubule based vesicle motility
ADCY5	Adenylate cyclase type 5	Intracellular signaling by cAMP
ADRBK1	Beta-adrenergic receptor kinase 1	Phosphorylation of adrenergic receptors
AKT1	Protein kinase B	PI3K-signallingxxx
AL928970.15	N.N.	n.a.
ANKRD16	Ankyrin repeat domain-containing protein 16	Translational fidelity
AP2S1	AP-2 complex subunit sigma-1	Clathrin dependent endocytosis
ARHGAP28	Rho GTPase-activating protein 28	Cytoskeleton organization
ARL8A	ADP-ribosylation factor-like protein 8A	Axonal transport
ASMTL	N-acetylserotonin O-methyltransferase-like protein	Nucleotide metabolism
ATF4	Activating transcription factor 4	Transcription factor
BTBD2	BTB/POZ domain-containing protein 2	neurogenesis
BX890604.9	Uncharacterized protein ENSP00000370496 fragment	n.a.
CCDC130	Coiled-coil domain-containing protein 130	Spliceosome
CCDC85C	Coiled-coil domain-containing protein 85C	Cerebral Cortex Development
CD151	Tspan24	Endocytosis
CDK2AP2	Cyclin-dependent kinase 2-associated protein 2	Stem cell proliferation
CDKN2C	Cyclin-dependent kinase 4 inhibitor C	Cell proliferation
CPE	Carboxypeptidase E precursor	Neuropeptide synthesis
DLL3	Delta-like protein 3 precursor	Neurogenesis
DOM3Z	Dom-3 homolog Z	RNA-modification, decapping
EF1A2	Elongation factor 1-alpha 2	Translation regulation
EFS	Embryonal Fyn-associated substrate	Cytosekeleton, cell migration
ERP29	Endoplasmic reticulum protein ERp29 precursor	n.a.
FADD	FAS-associated death domain protein	Apoptosis signaling pathway
FAM161A	Protein FAM161A	Microtubular organization, cilium formation
FEZF2	Fez family zinc finger protein 2	Dendritic arborization and spine formation
FST	Follistatin	Cell differentiation, embryonic development
FSTL3	Follistatin-related protein 3	Cell differentiation, embryonic development
FUS	RNA-binding protein FUS	spine formation, RNA stability, synapse stability
FVT1	Follicular lymphoma variant translocation 1	Sphingolipid synthesis
GDI2	Rab GDP dissociation inhibitor beta	GDP/GTP exchange reaction of Rab proteins
GIYD2	Structure-specific endonuclease subunit SLX1	DNA-repair
GPD1	Glycerol-3-phosphate dehydrogenase, cytoplasmic	Gluconeogenesis
GPR173	G protein coupled receptor 173	Neuron migration, gonadotropin signaling
GRAP2	GRB2-related adapter protein 2	T cell receptor signaling
GUSBP3	Putative Inactive Beta-Glucuronidase-Like Protein SMA3	Mucopolysaccharide degradation
HDAC10	Polyamine deacetylase HDAC10	Histone deacetylase activity
HINT1	Histidine triad nucleotide-binding protein 1	Purine nucleotide modification
HNRNPAB	Heterogeneous nuclear ribonucleoprotein A/B	Transcription reg., Epith.-Mesench.-Transform.
HSP90AB1	Heat shock protein HSP 90-beta	Chaperone function
IGFBP7	Insulin-like growth factor-binding protein 7	Modulation of growth factor actions
KIF18B	Kinesin-like protein KIF18B	Microtubule polymerization, mitosis
KIF5B	Kinesin-like protein KIF5B	Microtubular motor protein
MAFF	Transcription factor MafF	Transcription, embryonic development
MAP1LC3A	Microtubule-associated proteins 1A/1B light chain 3A	Microtubule binding, autophagosome formation
MAZ	Myc-associated zinc finger protein	Transcription factor-binding
MINDY3	Ubiquitin carboxyl-terminal hydrolase MINDY-3	Apoptosis
MLLT6	Protein AF-17	Transcription factor, ion flux
MRPL28	39S ribosomal protein L28, mitochondrial	Mitochondrial translation
MRPS11	28S ribosomal protein S11, mitochondrial	Mitochondrial translation
MRPS24	28S ribosomal protein S24, mitochondrial	Mitochondrial translation
NCAN*	Neurocan	Extracellular matrix, neural development
NCKIPSD	NCK-interacting protein with SH3 domain	Actin cytoskeleton, neurite formation
NDRG4	N-myc downstream-regulated gene 4 protein	Brain development
NFIX	Nuclear factor 1 X-type	Transcription factor
NPDC1	Neural proliferation differentiation and control protein 1	Neural stem cell proliferation
NUBP2	Nucleotide-binding protein 2	Neurite formation
NUDT16	U8 snoRNA-decapping enzyme	RNA-decapping enzyme
PGLS	6-phosphogluconolactonase	Pentose phosphate shunt
PI4KB	Phosphatidylinositol 4-kinase beta	Intracellular signaling
PIN1	Peptidyl-prolyl cis-trans isomerase NIMA-interacting 1	Protein isomerization
PIP5K1C	Phosphatidylinositol 4-phosphate 5-kinase type-1 gamma	Intracellular signaling
PITPNM1	Membrane-assoc. phosphatidylinositol transfer protein 1	Cytoskeleton, brain development
PLCB2	Phospholipase C-beta-2	Intracellular signaling
PLXNA2	Plexin-A2	Semaphorin receptor, axon guidance
PTBP1	Polypyrimidine tract-binding protein 1	pre-mRNA-splicing
PTK7	Inactive tyrosine-protein kinase 7	Wnt-signaling, axis elongation
RAB11B	Ras-related protein Rab-11B	Intracellular vesicle trafficking, autoimmunity
RAI1	Retinoic acid-induced protein 1	Transcription, Circadianic clock, Neurodvelopm.
RASD1	Dexamethasone-induced Ras-related protein 1	Transcription factor
RASL10A	Ras-like protein family member 10A	G Protein, intracellular signaling
RBFOX3	RNA binding protein fox-1 homolog 3	Splicing regulator, neuronal development
RNF10	RING finger protein 10	Transcription factor, myelination
RP11-706O15.1	HCG1981372, isoform CRA_c	Synaptic regulation
RPRM	Protein reprimo	Cell cycle arrest
RPS27	40S ribosomal protein S27	Translation
SDC3	Syndecan-3	Cell migration
SELO	Selenoprotein O	Protein adenylation
SEMA3B	Semaphorin-3B	Axon guidance
SEMA6C	Semaphorin-6C	Axon guidance
SLC17A7*	Vesicular glutamate transporter 1	Presynaptic glutamate reuptake
SPEF1	Sperm flagellar protein 1	Cell migration
SREBF1	Sterol regulatory element-binding protein 1	Transcription activator, lipid metabolism
STMN4*	Stathmin-4	Microtubular, neurite formation
STUB1	E3 ubiquitin-protein ligase CHIP	Protein ubiquitination
SULT1A1	Sulfotransferase 1A1	Catecholamine metabolism
SYT5*	Synaptotagmin 5	Synaptic vesicle recycling
TACC2	Transforming acidic coiled-coil-containing protein 2	Cortical neural progenitor proliferation
TFPI2	Tissue factor pathway inhibitor 2	Extracellular matrix
THAP10	THAP domain-containing protein 10	Transcription factor
TLCD2	TLC domain-containing protein 2	Regulation of membrane fluidity
TMEM254	Transmembrane protein 254	n.a.
TOMM40	Mitochondrial import receptor subunit TOM40 homolog	Mitochondrial matrix import
TUBB4	Tubulin beta-4A chain	Cytoskeleton, Microtubular polymerization
UBC	Polyubiquitin-C	Ubiquitinylation
UFC1	Ubiquitin-fold modifier-conjugating enzyme 1	Ubiquitinylation, Brain development
UQCRFS1	Cytochrome b-c1 complex subunit Rieske, mitochondrial	Respiratory chain functioning
USP3	Ubiquitin carboxyl-terminal hydrolase 3	Histone deubiquitination
WASHC1	WASH complex subunit 1	Intracellular vesicle transport
ZMAT2	Zinc finger matrin-type protein 2	mRNA splicing
ZNF133	Zinc finger protein 133	Transcription factor
ZNF553	Zinc finger protein 553	Transcription factor
ZNF692	Zinc finger protein 692	Transcription factor

Known schizphrenia candidates are labeled by an asterisk (*)

**Table 3:**
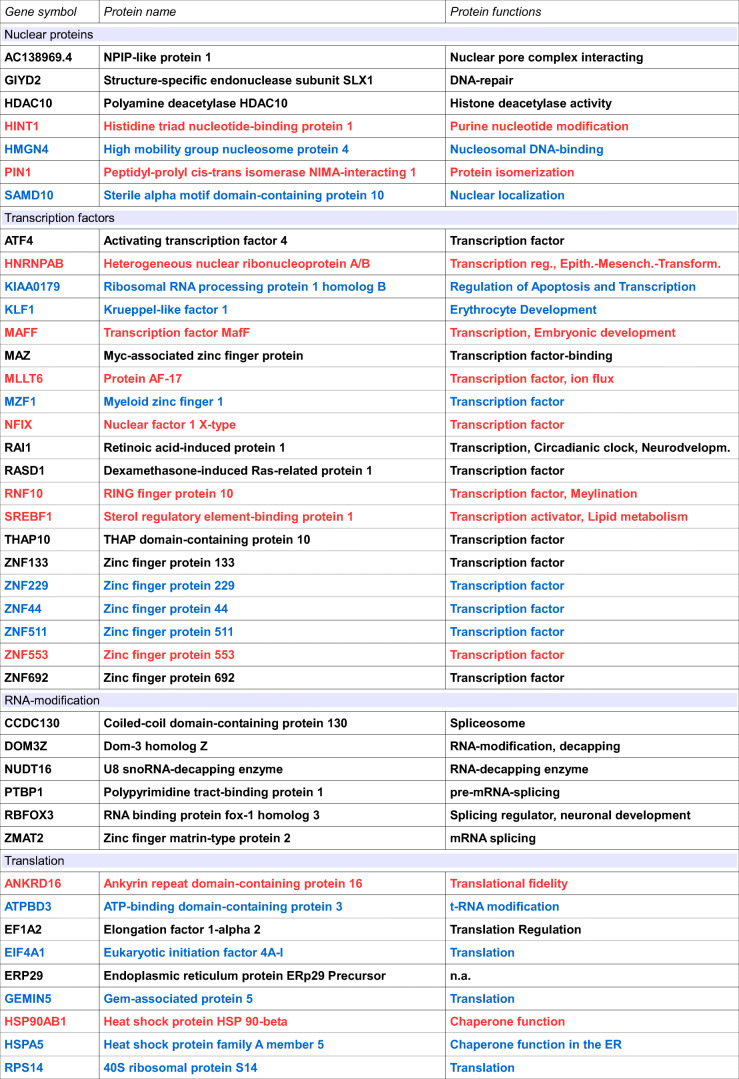

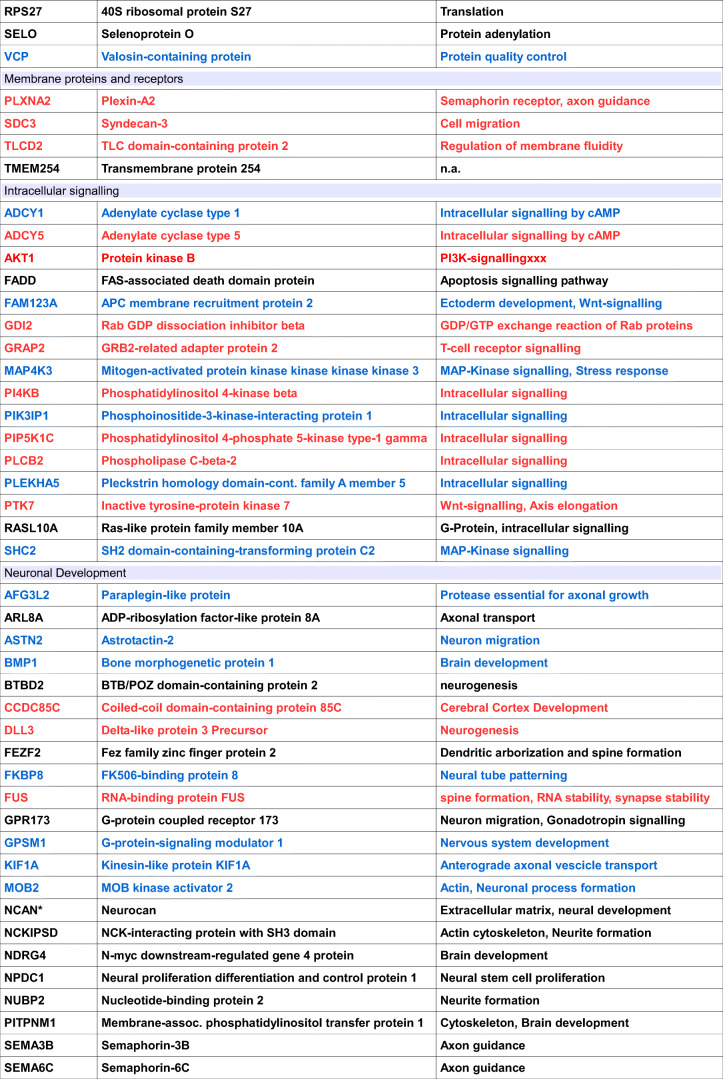

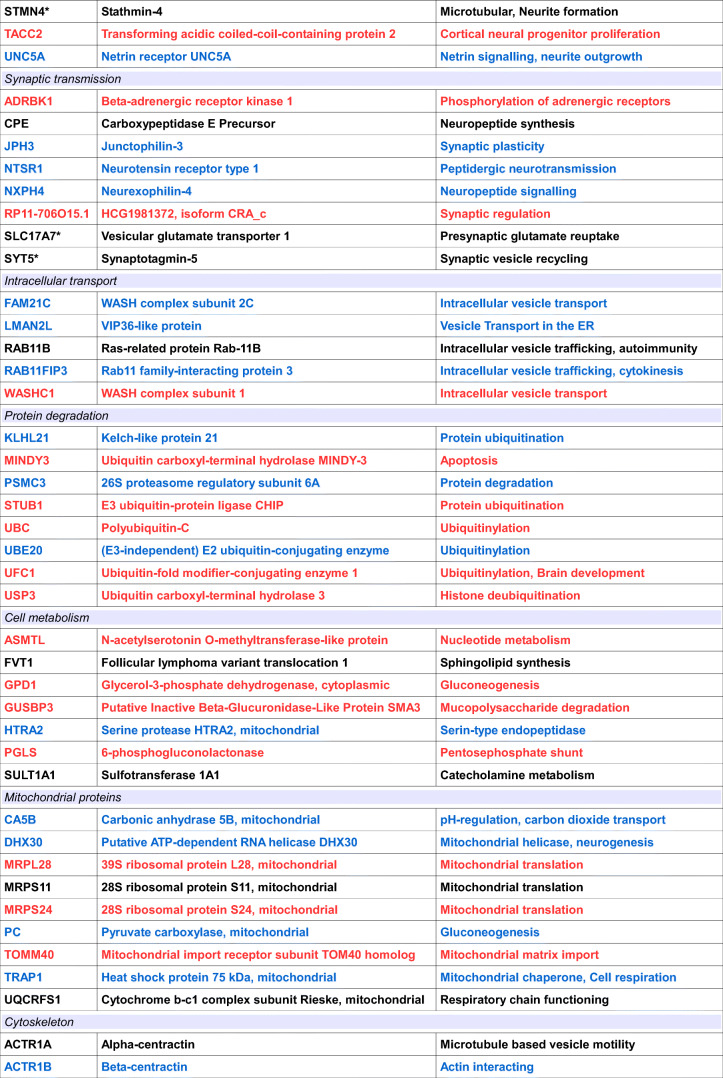

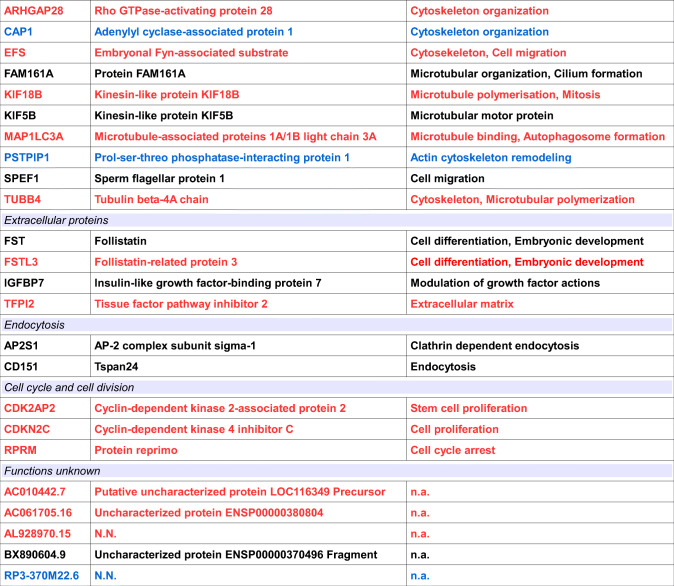
Proteins interacting with an antiserum directed to either ***Helicobacter pylori*** **(blue)** or ***Campylobacter jejuni*** **(red)**, or to **both bacteria (black)**

Proteins have been sorted according to their primary functions. Known Schizphrenia candidates are labeled by an asterisk (*)

**Fig. 3:**
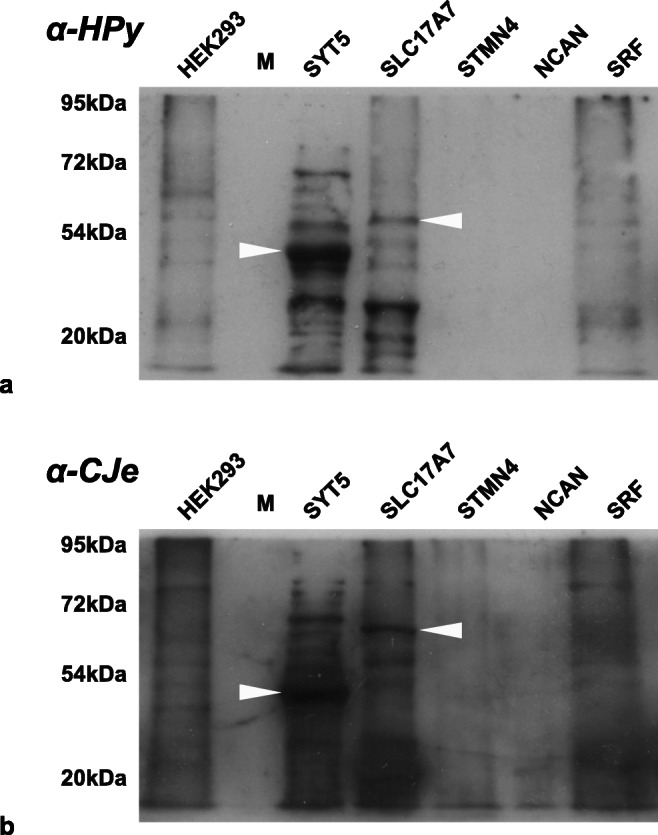
Western blot analysis of the cross-reactivity of antibodies directed to *Helicobacter pylori* (α-*HPy*) and *Campylobacter jejuni* (α-*CJe*) with different protein samples as provided by commercial HEK-293 overexpression lysates. **a** Cross-reactivity of α-*HPy* as revealed by a distinct immunopositive band can be observed for Syt5 and Vglut1 (Slc17a7), whereas Stmn4 and Ncan reveal no such band. Also a control lysate of non-transfected HEK293 cells, as well as an overexpression lysate of Srf is negative. **b** For α-*CJe* cross-reactivity as revealed by a distinct immunopositive band can be observed for Syt5 and Vglut1, whereas also in this case Stmn4 and Ncan reveal no such band. Again a control lysate of non-transfected HEK293 cells, as well as an overexpression lysate of Srf is negative. A corresponding negative control incubated with secondary antibodies only is shown in Supplementary Fig. 2

In a next step, we tried to confirm several of the protein interactions revealed by the MPA with an independent method. For this we tested interactions of α-*HPy* with a set of commercial overexpression lysates of these proteins in HEK293 cells, by Western blotting. As shown in Fig. 3a, α-*HPy* revealed again strong cross-reactivity with Syt5 and a somewhat weaker labeling of SLC17A7, demonstrating distinct immunoreactive bands at the appropriate molecular weight of ~ 43 kDa for Syt5 and ~ 62 kDa for Slc17a7. In contrast to this, interactions with other proteins showing cross-reactivity on the hexSelect MPA like Stmn4 or Ncan could not be confirmed by Western blot analysis with commercial overexpression lysates of these proteins. Also the totally unrelated protein Srf revealed no interaction with this antiserum. In a similar manner cross-reactivity of the closely related antiserum α-*CJe* with the same HEK293 overexpression lysates was also analyzed by Western blotting. As shown in Fig. 3b, α-*CJe* revealed an even stronger cross-reactivity with Syt5 and with SLC17A7, demonstrating distinct immunoreactive bands at the appropriate molecular weight of ~ 43 kDa for Syt5 and ~ 62 kDa for Slc17a7. Again, interactions with Stmn4 and Ncan could not be confirmed by Western blot analysis with a commercial overexpression lysate of this protein, and also the totally unrelated protein Srf revealed no interaction with this antiserum. Again for control purposes, a similar Western blot was incubated with secondary antibodies only, revealing only a slight background staining (see Supplementary Fig. 2).

**Fig. 4:**
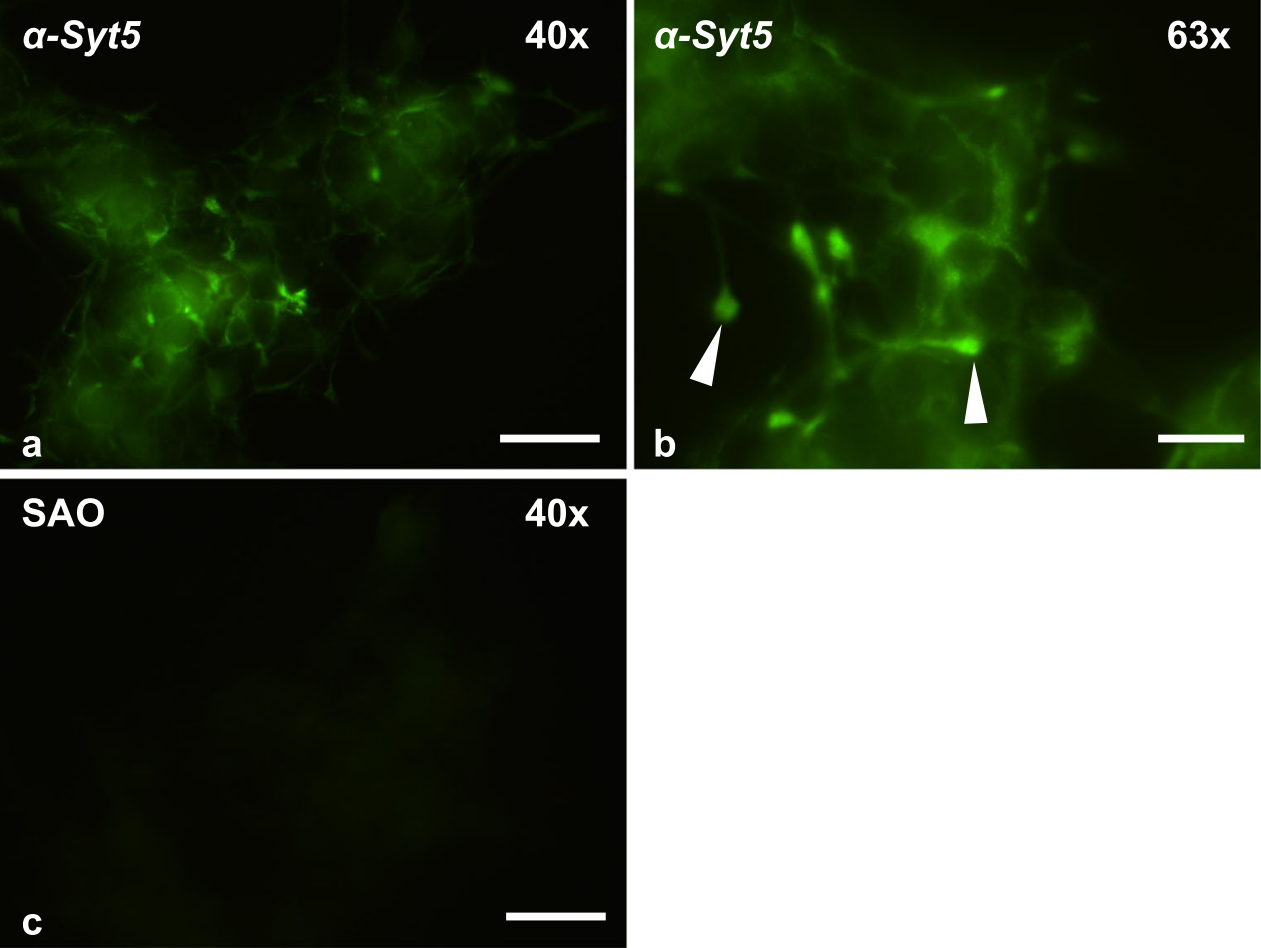
Expression of synaptotagmin 5 (Syt5) in SiMa neuroblastoma cells as revealed by indirect immunofluorescence. **a** Fluorescence image of an immune incubation of SiMa neuroblastoma cells with a polyclonal antiserum directed to Syt5 at a lower magnification (× 40). **b** Higher magnification (× 63) fluorescence image of an immune incubation of SiMa neuroblastoma cells with a polyclonal antiserum directed to Syt5, revealing a distinct labeling of synaptic terminals (arrowheads). **c** Lower magnification (× 40) fluorescence image of a culture of SiMa neuroblastoma cells incubated with the secondary antibody only (SAO) revealing no staining at all. Bars in (**a**) and (**c**) = 20 μm, Bar in (**b**) = 10 μm

**Fig. 5:**
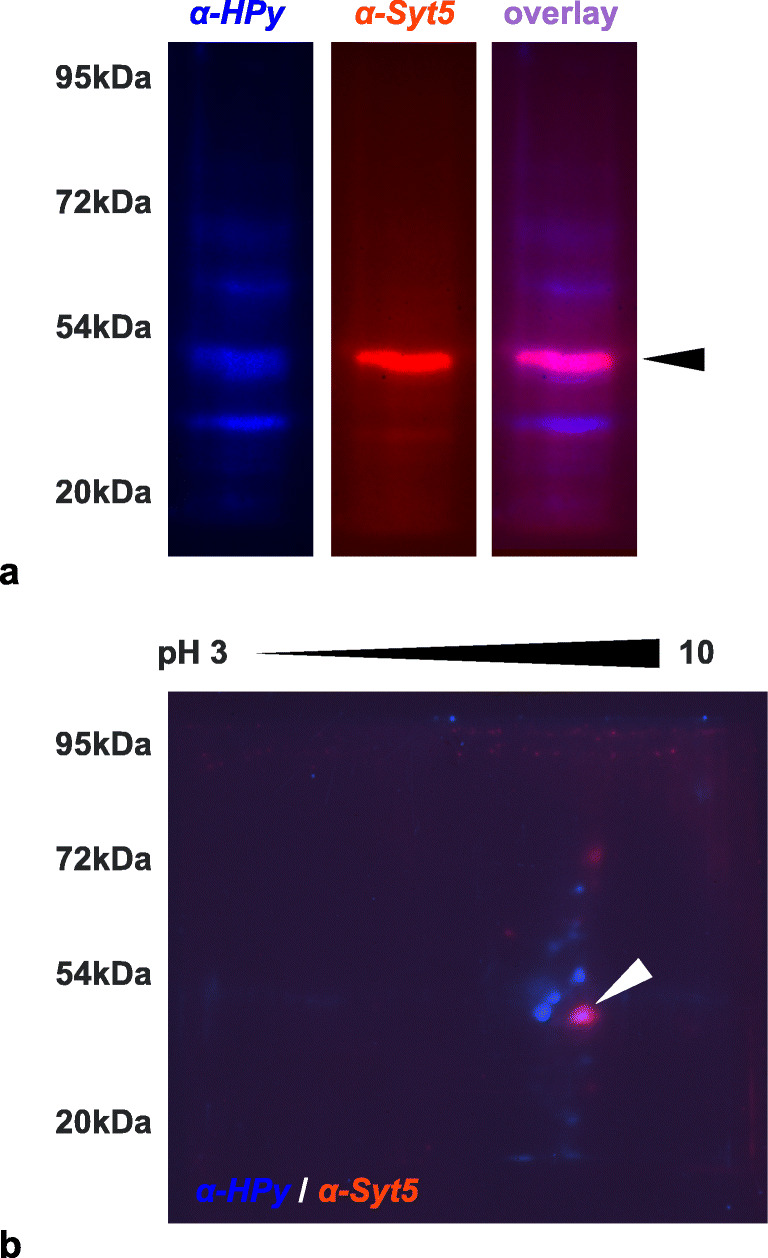
Identification of synaptotagmin 5 (Syt5) as an interaction partner of α-*HPy* in SiMa human neuroblastoma cells as revealed by one- and two-dimensional Western blot analysis. **a** Standard Western blot analysis of a whole cell protein extract of SiMa cells incubated with either α-*HPy* (blue) or α-Syt5 (red) revealing in both cases a distinct immunoreactive band with a molecular weight of ~ 43 kDa the pink staining in the overlay image reveals them to be identical. **b** Overlay image of a two-dimensional Western blot analysis of a whole cell protein extract of SiMa cells incubated first with α-*HPy* (blue) and after stripping with α-Syt5 (red) revealing an immunoreactive spot with a molecular weight of ~ 43 kDa and an isoelectric pH of ~ 9.3, with a distinct co-labeling as revealed by its pink color. A corresponding negative control incubated with secondary antibodies only is shown in Supplementary Fig. 3

**Fig. 6:**
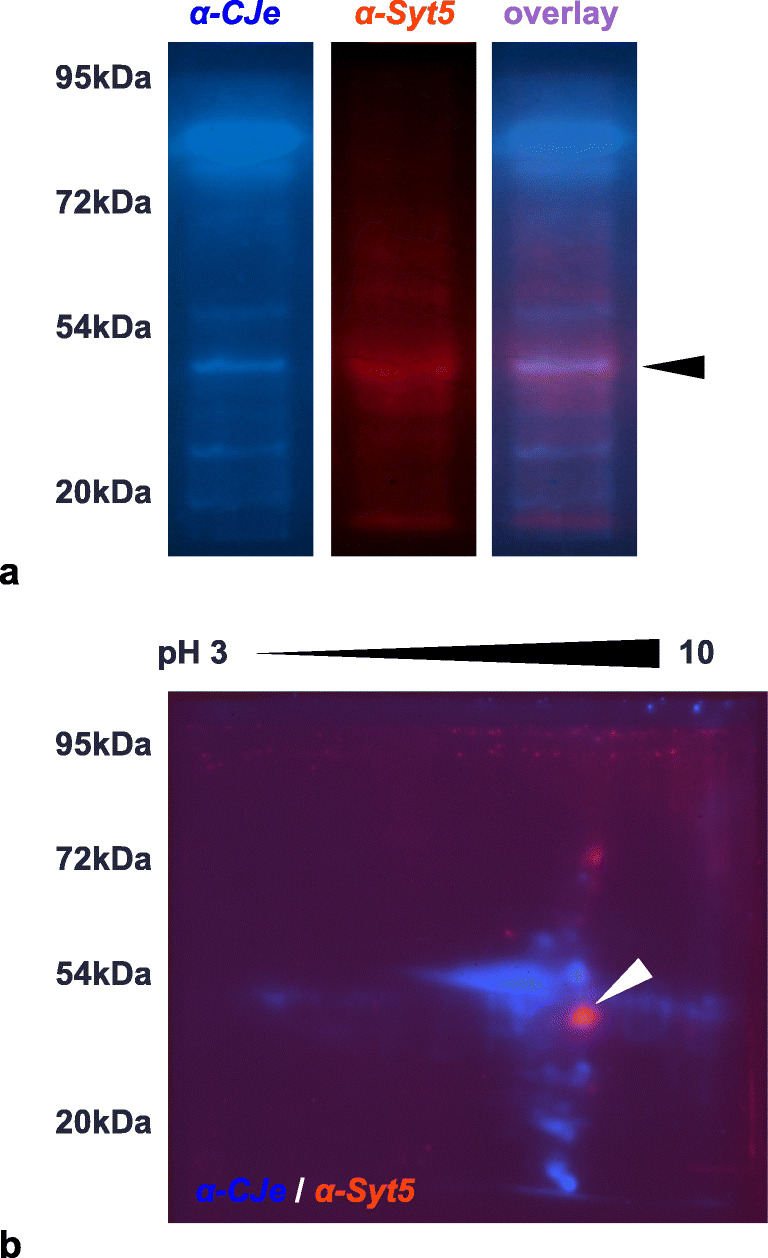
Identification of synaptotagmin 5 (Syt5) as an interaction partner of α-*CJe* in SiMa human neuroblastoma cells as revealed by one- and two-dimensional Western blot analysis. **a** Standard Western blot analysis of a whole cell protein extract of SiMa cells incubated with either α-*CJe* (blue) or after stripping with α-Syt5 (red) revealing in both cases a distinct immunoreactive band with a molecular weight of ~ 43 kDa, the pink staining in the overlay image reveals them to be identical. **b** Overlay image of a two-dimensional Western blot analysis of a whole cell protein extract of SiMa cells incubated first with α-*CJe* (blue) and after stripping with α-Syt5 (red) revealing an immunoreactive spot with a molecular weight of ~ 43 kDa and an isoelectric pH of ~ 9.3 with a distinct co-labeling as revealed by its pink color. A corresponding negative control incubated with secondary antibodies only is shown in Supplementary Fig. 4

Due to its prominent role in exocytosis and synaptic transmission, and its exclusive expression in the human brain with high abundance in frontal cortex and the amygdala, we selected the interactions of α-*HPy* and α-*CJe* with Syt5 for a more detailed functional analysis. For this, we used here SiMa cells, a human neuroblastoma cell line with a neuronal phenotype as revealed by the formation of neurites and of cholinergic synapses. As shown in Figs. 4, 5, and 6, we clarified first whether these cells expressed Syt5, using immunocytochemistry (Fig. 4) and both standard and 2D-Western blot analysis (Figs. 5 and 6). Thus, immunofluorescent labeling revealed SiMa cells to express Syt5 in synaptic boutons at their neurite terminals (Fig. 4). Further along this line, in a standard Western blot (Fig. 5a), a whole cell protein extract of SiMa cells, incubated with a polyclonal antibody to Syt5 (α-Syt5) revealed a distinctly labeled protein band at ~ 43 kDa. After stripping and reincubation of the same blotting membrane with α-*HPy*, a similar band was labeled, and finally, the false color overlay image revealed both bands to run at the same size (Fig. 5a). Similarly the false color overlay image of a 2D-Western blot analysis of a SiMa cell whole cell extract (Fig. 5b) revealed α-Syt5 to label a protein spot of ~ 43 kDa with a characteristic isoelectric point of pH ~ 9.3. Again, after stripping and reincubation with α-*HPy*, the same protein spot was labeled (Fig. 5b). Similarly, as shown in Fig. 6, another standard Western blot with a whole cell protein extract of SiMa cells (Fig. 6a), a polyclonal antibody to Syt5 (α-Syt5), labeled again a specific protein band of ~ 43 kDa, and in this case after stripping and reincubation of the same blotting membrane with α-*CJe*, a protein band of the same size was labeled which in the false color overlay image turned out to be identical to the one labeled by α-Syt5 (Fig. 6a). Also in this case the false color overlay image of a 2D-Western blot analysis of a SiMa cell whole cell extract (Fig. 6b) revealed α-Syt5 to label a protein spot of ~ 43 kDa with a characteristic isoelectric point of pH ~ 9.3, and after stripping and reincubation with α-*CJe*, the same protein spot was labeled (Fig. 6b). Also in this case, control experiments were performed by incubating similar Western blots with secondary antibodies only, revealing again only a slight background staining (see Supplementary Figs. 3 and 4).

In order to further confirm both, expression of Syt5 in SiMa neuroblastoma cells and interactions of α-*HPy* and α-*CJe* with Syt5 in these cells, a-gene specific knockdown of the Syt5 mRNA was performed by transfecting these cells with a commercial Syt5 shRNA expression vector (Supplementary Fig. 5). As revealed by the Western blot analysis for Syt5 (Supplementary Fig. 5a), immunoreactivity for this protein was distinctly decreased in the whole cell protein extract from SiMa neuroblastoma cells transfected with the Syt5 shRNA expression vector, as compared with untreated cells, and also to cells transfected with a non-mammalian shRNA expression vector for control purposes. Likewise a similar Western blot, however in this case incubated with α-*HPy* (Supplementary Fig. 5b) revealed a distinctly decreased staining of the protein band representing interactions of this antiserum with Syt5. Incubation of the same Western blot with an antibody directed to β-actin confirmed the amount of protein loaded on each lane to be identical. The same was true for α-*Syt5* and α-*CJe*, since also in this case a distinct reduction in immunoreactivity for both α-*Syt5* (Supplementary Fig. 5d) and for the protein band representing the interactions of α-*CJe* with Syt5 (Supplementary Fig. 5e) could be observed. Also in this case incubation of the same Western blot with an antibody directed to β-actin confirmed the amount of protein loaded on each lane to be identical.

**Fig. 7:**
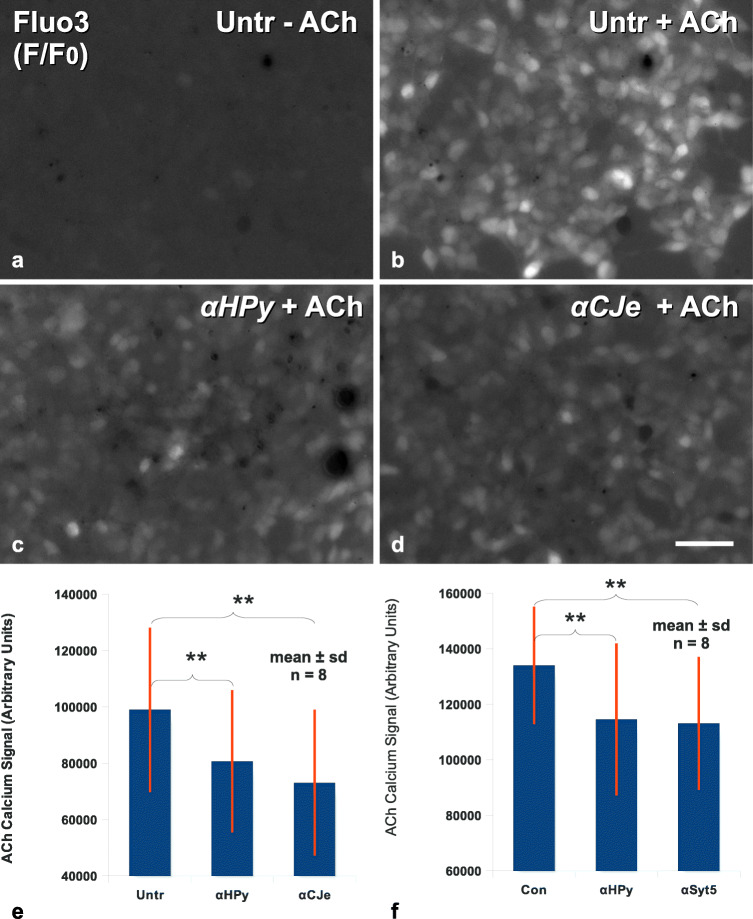
Effects of the antibacterial antisera α-*HPy*, and α-*CJe* on acetylcholine (ACh) dependent calcium transients in cultures of SiMa human neuroblastoma cells, as revealed by imaging with the calcium sensitive fluorescent dye Fluo3-AM. **a** Image of the maximum plot of the fluorescence intensity (F/F_0_) of a non-treated control culture of SiMa human neuroblastoma cells revealing weak changes in the intracellular calcium concentration of different cells, representing the normal background activity. **b** Image of the maximum plot of the fluorescence intensity (F/F_0_) of a non-treated control culture of SiMa human neuroblastoma cells, however in this case perfused for 50 s with 10 nmol/l of ACh in standard bath solution, revealing a distinct increase in intracellular calcium concentration. **c** Image of the maximum plot of the fluorescence intensity (F/F_0_) of a Fluo3-stained culture of SiMa human neuroblastoma cells, pretreated for 12 h with 10 μg/ml of α-*HPy*, and subsequently perfused for 50 s with 10 nmol/l of ACh in standard bath solution. In this case the ACh-dependent increase in intracellular calcium concentration as revealed by Fluo3-AM is distinctly weaker as compared with the control experiment shown in (**b**). **d** Image of the maximum plot of the fluorescence intensity (F/F_0_) of a Fluo3-stained culture of SiMa human neuroblastoma cells pretreated for 12 h with 10 μg/ml of α-*CJe*, and subsequently perfused for 50 s with 10 nmol/l of ACh in standard bath solution. Also in this case the ACh-dependent increase in intracellular calcium concentration as revealed by Fluo3-AM is distinctly weaker as compared with the control experiment shown in (**b**). **e** Diagram of the statistical evaluation of a series of experiments as shown in (**a**–**d**), revealing the reduction in the intensity of ACh-dependent calcium transients of human SiMa neuroblastoma cells pretreated for 12 h with either 10-μg/ml α-*HPy* or α-*CJe* to be highly significant. **f** Diagram of the statistical evaluation of a similar series of experiments as shown in (**a**–**d**), however in this case pretreated with α-*HPy* or α-Syt5, revealing the equally observable reduction in the intensity of ACh-dependent calcium transients of human SiMa neuroblastoma cells pretreated for 12 h with either 10 μg/ml α-*HPy* or α-Syt5 in comparison with control cultures, to be highly significant. **p < 0.01; Bar in (**d**) = 50 μm

Syt5 has already been shown to play a role in Ca^++^-dependent synaptic transmission. As many other neuroblastoma cell lines, SiMa cells are known to express also receptors for acetylcholine (ACh), ligand binding of which is able to elicit intracellular Ca^++^-transients. In order to investigate possible functional consequences of α-*HPy* binding to Syt5, we analyzed now, whether α-*HPy* was able to interfere with synaptic activity as revealed by ACh-dependent Ca^++^-transients in this cell line. As revealed in Fig. 7, control cells stained with the Ca^++^-dependent fluorescent dye Fluo3-AM (Fig. 7a, b) revealed a strong transient intracellular calcium signal (F/F_0_) upon stimulation with 10-nmol/l ACh. In contrast to that in cells pretreated for 12 h with 10-μg/ml α-*HPy* and then stained with the Ca^++^-dependent fluorescent dye Fluo3-AM (Fig. 7c), the intensity of ACh-dependent intracellular Ca^++^-transients (F/F_0_) was distinctly reduced as compared with the control cultures. Likewise a pretreatment of SiMa cells with 10-μg/ml α-*CJe* (Fig. 7d) resulted also in a reduction of the intensity of ACh-dependent Ca^++^-transients as compared with control cultures. The statistical evaluation of a series of such experiments confirmed the effect of α-*HPy* on ACh-dependent Ca^++^-transients to be statistically significant (Fig. 7e). Statistical evaluation of a similar series of experiments, however in this case besides an incubation with α-*HPy* pretreated also with a polyclonal antiserum to Syt5, revealed a similar statistically significant effect of this latter antiserum on ACh-dependent Ca^++^-transients (Fig. 7e).

**Fig. 8:**
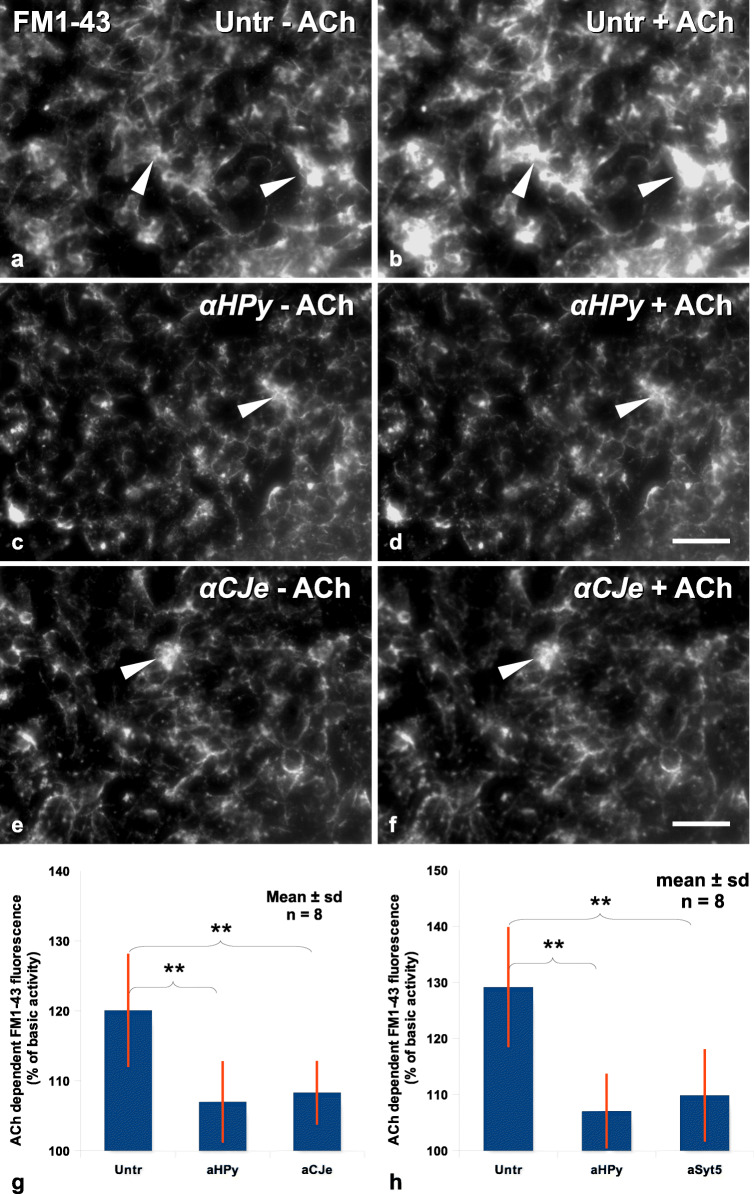
Effects of different antibacterial antisera (α-*HPy*, α-***CJe***) on ACh-stimulated exocytosis/vesicle recycling in cultures of SiMa human neuroblastoma cells, as visualized by life staining with the fluorescent vesicle marker FM1-43. **a** Fluorescence image of a non-treated control culture of SiMa human neuroblastoma cells, perfused for 50 s with 10 μmol/l of FM1-43 in standard bath solution, followed by 50 s with standard bath solution alone, revealing a distinct fluorescence signal in intracellular storage vesicles all over the cells. **b** Fluorescence image of the same cells as shown in (**a**), perfused again for 50 s with 10 μmol/l of FM1-43 in standard bath solution however in this case in the presence of 10 nmol/l ACh, (followed by 50 s with standard bath solution alone, revealing a distinct ACh-dependent increase in the fluorescence signal in the intracellular storage vesicles of these cells. **c** Fluorescence image of a culture of SiMa human neuroblastoma cells pretreated for 12 h with 10-μg/ml α-*HPy*, and then perfused for 50 s with 10 μmol/l of FM1-43 in standard bath solution, followed by 50 s with standard bath solution alone, revealing a distinct fluorescence signal in intracellular storage vesicles all over the cells. **d** Fluorescence image of the same cells as shown in (**c**), also perfused for 50 s with 10 μmol/l of FM1-43 in standard bath solution, however in this case in the presence of 10-nmol/l ACh, followed by 50 s with standard bath solution alone, revealing only a moderate ACh-dependent increase in the fluorescence signal in the intracellular storage vesicles of these cells. **e** Fluorescence image of a culture of SiMa human neuroblastoma cells pretreated for 12 h with 10-μg/ml α-*CJe*, and then perfused for 50 s with 10 μmol/l of FM1-43 in standard bath solution, followed by 50 s with standard bath solution alone, revealing a distinct fluorescence signal in intracellular storage vesicles all over the cells. **f** Fluorescence image of the same cells as shown in (**e**), also perfused for 50 s with 10 μmol/l of FM1-43 in standard bath solution, however in this case in the presence of 10 nmol/l ACh, followed by 50 s with standard bath solution alone, revealing again only a moderate ACh-dependent increase in the fluorescence signal in the intracellular storage vesicles of these cells. **g** Diagram of the statistical evaluation of a series of experiments as shown in (**a**–**f**), revealing the reduction in the ACh-dependent increase in FM1-43 fluorescence of human SiMa neuroblastoma cells pretreated for 12 h with either 10 μg/ml α-*HPy* or α-*CJe* to be highly significant. **h** Diagram of the statistical evaluation of a similar series of experiments as shown in (**a**–**f**), however in this case pretreated with α-*HPy* or α-Syt5, revealing the reduction in the ACh-dependent increase in FM1-43 fluorescence of human SiMa neuroblastoma cells pretreated for 12 h with either 10-μg/ml α-*HPy* or α-Syt5 as compared WITH control cultures to be highly significant. **p < 0,01; Bar in (**f**) = 50 μm

Chemical neurotransmission is characterized by the exocytotic release of neurotransmitters, followed by the reuptake and recycling of the vesicular membrane by clathrin-mediated endocytosis. Endocytotic vesicle recycling is therefore also an indicator of synaptic activity that can be visualized by the vesicular uptake and incorporation of the fluorescent dye FM1-43. We used this effect here to further characterize and quantify the effects of α-*HPy*, α-*CJe*, and α-Syt5 on ACh-dependent synaptic activity in SiMa neuroblastoma cells. As shown in Fig. 8, control cells incubated with FM1-43 in the absence of ACh revealed a distinct increase in FM1-43 incorporation upon stimulation with 10-nmol/l ACh (Fig. 8a, b). In cells pretreated for 12 h with 10-μg/ml α-*HPy* and then incubated with FM1-43 (Fig. 8c), the level of ACh-dependent uptake of FM1-43 was distinctly reduced as compared with the control cultures. Likewise a pretreatment of SiMa cells with 10-μg/ml α-*CJe* (Fig. 8d) resulted also in a reduction of ACh-dependent incorporation of FM1-43 as compared with control cultures. The statistical evaluation of a series of such experiments confirmed the effect of α-*HPy* on ACh-dependent incorporation of FM1-43 in SiMa cells to be statistically significant (Fig. 8e). Statistical evaluation of a similar series of experiments, however in this case besides an incubation with α-*HPy* pretreated also with a polyclonal antiserum to Syt5 (α-Syt5), revealed a similar statistically significant effect of both antisera on ACh-dependent uptake of FM1-43 in SiMa cells (Fig. 8e).

**Fig. 9:**
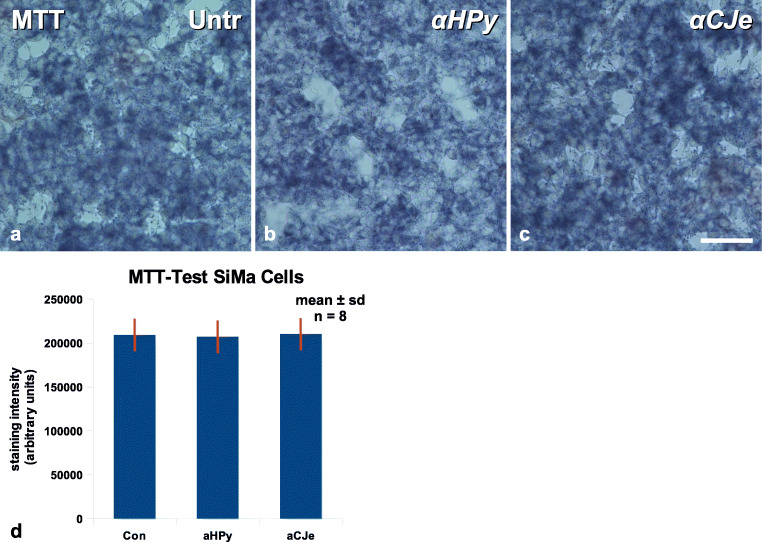
Effects of different antibacterial antisera (α-*HPy*, α-*CJe*) on staining of SiMa neuroblastoma cells with the metabolic marker dye 3-(4,5-Dimethyl-2-thiazolyl)-2,5-diphenyl-2H-tetrazolium bromide (MTT). **a** Untreated control culture of SiMa human neuroblastoma cells, revealing the typical staining pattern for this dye. **b** Culture of SiMa human neuroblastoma cells, pretreated for 12 h with 10 μg/ml of α-*HPy*, which shows an almost similar MTT staining intensity as in control treated cells. **c** Culture of SiMa human neuroblastoma cells, pretreated for 12 h with 10 μg/ml of α-*CJe*, revealing an almost similar MTT staining intensity as control treated cells. **d** Diagram of the statistical evaluation of a series of experiments as shown in (**a**–**c**), revealing no significant changes in MTT-staining and thus in cell viability of human SiMa neuroblastoma cells pretreated for 12 h with either 10 μg/ml of α-*HPy* or α-*CJe*, as compared to untreated controls. Bar in (**c**) = 40 μm

**Fig. 10:**
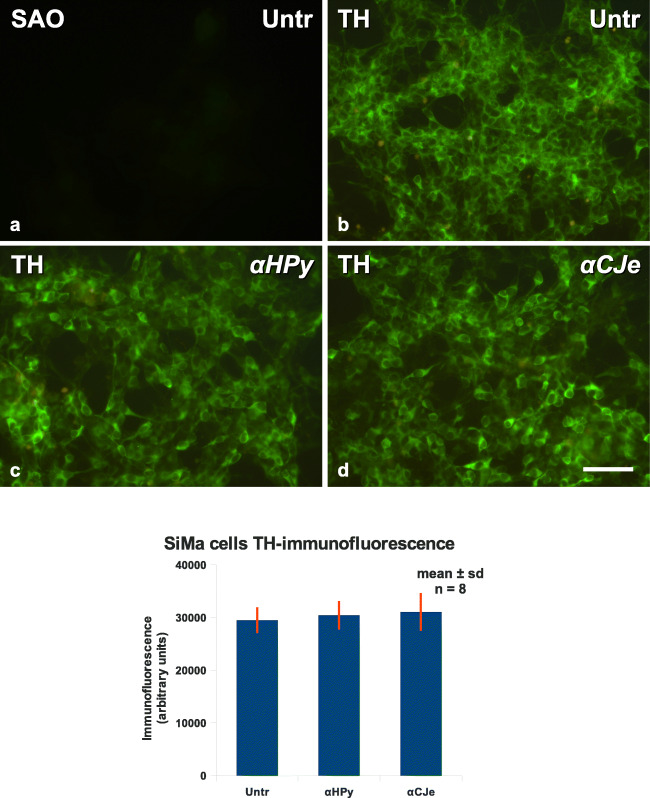
Effects of different antibacterial antisera (α-*HPy*, α-*CJe*) on expression of the dopaminergic marker protein tyrosine hydroxylase (TH) in cultures of SiMa human neuroblastoma cells, as visualized by indirect immunofluorescent staining. **a** Fluorescence image of an untreated control culture of SiMa human neuroblastoma cells (Untr) stained in this case after fixation with the secondary antibody only (SAO), revealing only a very weak background staining. **b** Fluorescence image of an untreated control culture of SiMa human neuroblastoma cells, in this case incubated after fixation with an antibody specific for tyrosine hydroxylase (TH), revealing an intense cytoplasmic staining in the cells. **c** Fluorescence image of a culture of SiMa human neuroblastoma cells, pretreated for 12 h with 10 μg/ml of α-*HPy*, which after fixation were stained for TH, revealing an intense cytoplasmic labeling in most of the cells. **d** Fluorescence image of a culture of SiMa human neuroblastoma cells, pretreated for 12 h with 10 μg/ml of α-*CJe*, and after fixation stained for TH, revealing an intense cytoplasmic labeling in most of the cells. **e** Diagram of the statistical evaluation of a series of experiments as shown in (**a**–**d**), revealing no significant differences in TH-fluorescence in human SiMa neuroblastoma cells pretreated for 12 h with either 10-μg/ml α-*HPy* or α-*CJe*. Bar in (**d**) = 40 μm

In order to exclude that the effects of the antibacterial antibodies on SiMa neuroblastoma cells revealed in the present study were not only the result of impaired cell metabolism, an MTT assay for cell viability was performed. As presented in Fig. 9, control cells incubated with MTT revealed the typical mitochondrial staining pattern in almost all cells (Fig. 9a). A similar MTT staining, both with regard to intensity and distribution, was obtained also in SiMa cells pretreated for 12 h with either 10-μg/ml α-*HPy* (Fig. 9b) or 10-μg/ml α-*CJe* (Fig. 9c). As revealed by the statistical evaluation of a series of such experiments, a 12-h pretreatment of SiMa cells with either α-*HPy* or α-*CJe* was not able to cause significant differences in MTT staining intensity (Fig. 9d).

Further along this line, expression of tyrosine hydroxylase, an established marker for neuroblastoma cells, was investigated by immunofluorescent staining. As revealed in Fig. 10, SiMa cells incubated with only the secondary antibody (SAO) exhibited almost no fluorescent staining (Fig. 10a). In contrast, cells incubated in addition with an antibody to tyrosine hydroxylase revealed a typical cytoplasmic staining (Fig. 10b). A similar staining, both with regard to intensity and distribution, was also obtained for SiMa cells pretreated for 12 h with either 10-μg/ml α-*HPy* (Fig. 10c) or 10-μg/ml α-*CJe* (Fig. 10d). As revealed by the statistical evaluation of a series of such experiments, a 12-h pretreatment of SiMa cells with either α-*HPy* or α-*CJe* was not able to cause significant differences in tyrosine hydroxylase immunoreactivity (Fig. 10e).

## Discussion

Prenatal maternal bacterial infections have been reported to play a role in the outbreak of psychosis in the offspring later in life (Babulas et al. 2006; Sørensen et al. 2009). Thus, gonococcal infections seem to be associated with an increased schizophrenia risk; however, upper respiratory tract infections as well as ear-nose-throat infections also seem to have a similar effect (Sørensen et al. 2009). In a previous study (Almamy et al. 2017), we have already demonstrated that polyclonal antisera directed to *Neisseria gonorrhoeae* are indeed able to interact with different neuronal proteins, among which several schizophrenia candidate proteins can be found (Almamy et al. 2017). In the present study, we have now extended these findings on interaction partners and functional effects of antisera to the microaerophilic gastric bacterium *Helicobacter pylori* (*HPy*) and the closely related intestinal tract bacterium *Campylobacter jejuni* (*CJe*), which have also been implicated as a risk factor for schizophrenia pathology (de Hert et al. 1997; Yilmaz et al. 2008). By this we were able to document for the first time an interaction of polyclonal antisera directed to both *HPy* and *CJe* with the human synaptic calcium sensor synaptotagmin 5 (Syt5), and could correlate this interaction in SiMa human neuroblastoma cells to functional changes such as a diminished capability to respond to acetylcholine (ACh) with intracellular Ca^++^-transients, and a diminished rate of ACh-dependent vesicle recycling.

A possible mechanism underlying such an effect could be molecular mimicry, consisting of the induction of antibacterial or antiviral antibodies as a result of an infection, and, due to molecular similarities, an erroneous interaction of these antibodies with cellular proteins, functions of which can be impaired by antibody binding (Oates et al. 1977; Oldstone 1998). The present study tested this hypothesis by investigating interaction partners of antisera to *HPy* (α-*HPy*) and *CJe* (α-*CJe*) on a commercial multiprotein array and was able to identify comparably high numbers of different proteins with the ability to bind these antibodies. Thus, for both α-*HPy* and α-*CJe* proteins, interacting with these antisera belonged to a wide variety of functional groups including nuclear proteins and transcription factors such as Hdac10 and Atf4, transcriptional and translational regulators like NUDT16 and RPS27, membrane receptors and intracellular signaling molecules like Gpr173 and AKT1, proteins involved in intracellular transport and protein degradation like Rab11b and several ubiquitinylation factors, proteins involved in cell metabolism like Fvt1 and Sult1a1, mitochondrial proteins like Mros11 and Uqcrfs1, cytoskeletal regulators like Actr1a and Kif5b, extracellular signaling molecules like Fst and Ncan, as well as proteins involved in endocytosis like Cd130. Finally, a large group of proteins interacting with α-*HPy* and α-*CJe* comprised factors involved in neural development and synaptic transmission such as Npdc1 and Syt5.

Not all proteins with antibacterial cross-reactivity revealed affinity to both of the investigated antisera, such that of a total number of 153 identified interaction partners, only 53 bound to both antisera (α-*HPy* and α-*CJe*), whereas 46 and 54 proteins reacted with either α-*HPy* or α-*CJe*, respectively. Proteins with affinity to only α-*HPy* and an importance for neurodevelopment include the neuron migration factor astrotactin-2 and the growth factor Bmp1, whereas for α-*CJe* the proteins Dll3 and Fus would be interesting candidates for further investigations. However, due to the multitude of interaction partners for α-*HPy* and α-*CJe* on the heXselect protein array, the selection of proteins with putative importance for neurodevelopment and schizophrenia pathology remains a difficult task. We tried to tackle this by selecting proteins with known relevance as schizophrenia candidate genes, or proteins with a role in neurodevelopment and high expression in schizophrenia-relevant brain regions like the frontal and/or cingulate cortex, as well as the amygdala. This led us to proteins like synaptotagmin 5 (Syt5; Maycox et al. 2009; Martins-de-Souza et al. 2010), vesicular glutamate transporter 1 (Slc17a7; Eastwood and Harrison 2005; Bitanihirwe et al. 2009), Stathmin-4 (English et al. 2009; Wang et al. 2010), and neurocan (Ncan; Schultz et al. 2014; Wang et al. 2016), which we have chosen for further analysis. By different Western blotting techniques, we could confirm at least some of the interactions demonstrated on the multiprotein array such as the interaction of α-*HPy* and α-*CJe* with Vglut-1 and Syt5, whereas the interactions of these antisera with either Stmn4 or Ncan as demonstrated on the multiprotein array could not be confirmed by Western blot analysis. This may be due to differences in protein conformation due to differences in the fixation procedures and the overall chemical environment. However, a much more difficult task remains to clarify the possible role of all the other proteins for schizophrenia pathology. Candidates with the highest possibility for a disease relevance will probably be those proteins located at or within the cell membrane, since they are most likely to get into direct contact with circulating immunoglobulins. However, also intracellular proteins could act as autoantigens, probably by T cell-mediated mechanisms (Iorio and Lennon 2012).

Due to its relevance in synaptic transmission, and its well-documented role for schizophrenia pathogenesis, for a functional analysis we focused in the present study on synaptotagmin 5. Syt5 is a member of a family of evolutionary conserved vesicle proteins (Südhof 1995), which is primarily expressed in the brain, playing a crucial role in calcium-regulated exocytosis of synaptic vesicles (Geppert et al. 1994; Mikoshiba et al. 1995). Hints for a role of Syt5 in schizophrenia pathology come from the fact that in rats treated with neuroleptic drugs, expression of Syt5 is reduced in the frontal cortex (Kontkanen et al. 2002). In addition, postmortem samples from different regions in a schizophrenic patient’s brain reveal distinct changes in Syt5 expression, including a reduction in Syt5 mRNA in the frontal cortex (Maycox et al. 2009), whereas in the thalamus Syt5 protein is increased (Martins-de-Souza et al. 2010). Also the comparably high expression levels of Syt5 mRNA in normal human prefrontal and cingulate cortex, and in the amygdala (see “http://biogps.org/#goto=genereport&id=6861” probe sets 206161_x_at, and 206162_x_at), are highly suggestive of a possible role for Syt5 in schizophrenia pathology.

Another important physiological process, for which Syt5 has been already shown to play a key role, is glucose-dependent release of insulin and glucagon in pancreatic island cells (Iezzi et al. 2004). This finding would also fit with a role of Syt5 in schizophrenia pathology, since insulin resistance and resulting type II diabetes is a long-known key feature of this disease even in drug-naïve patients (Collins 1957; Schimmelbusch et al. 1971). However, these early findings were covered for several decades by the more prominent effects of neuroleptic drugs, which are also able to increase the diabetes risk in schizophrenic patients (Melkersson et al. 1999; Lindenmayer et al. 2001). Only in recent years the significance of altered glucose metabolism in drug-naive schizophrenic patients has come again into the focus of scientific interest (Verma et al. 2009).

Towards a similar direction points the finding that Syt5 is involved in the regulation of calcium-dependent Weibel-Palade body exocytosis and with this in the secretion of von Willebrand factor by human vascular endothelial cells (Lenzi et al. 2019). Thus, antibodies interacting with Syt5 such as α-*HPy* and α-*CJe* could interfere with this process resulting in altered blood coagulation, a symptom that can be observed also in schizophrenic patients, where the concentration of vWF has already previously been demonstrated to be increased in the blood serum as revealed by ELISA (Hope et al. 2009). Further along this line, markers for a pathological activation of blood clotting and fibrinolysis, as well as activation of thrombocytes, have been previously demonstrated in unmedicated patients with acute psychosis (Masopust et al. 2011).

Immunological studies regarding human neuronal autoimmune disorders are always difficult to perform, since suitable model systems are hard to find. Especially investigations with the common laboratory animals like rats, mice or guinea pigs always suffer from the difficulty that regarding their immunoreactivity, differences between human and animal proteins exist. This turns the pathological effects observed difficult to compare. Even in primate animal models such as the marmoset monkey *Callithrix jacchus* (Reuss et al. 2016), a guarantee that the obtained results represent the conditions in the human brain cannot be given. In the present study, we therefore decided to use the human neuroblastoma cell line SiMa due to their numerous and long neurite-like processes with a clearly visible growth cone that seemed to be a fairly good compromise. Fortunately to us we were able to demonstrate expression of Syt5 in this cell line allowing us to use it for functional tests of the effects of α-*HPy* and α-*CJe* on ACh-dependent calcium transients and vesicle recycling. Independent support for the use of this neuroblastoma cell line in our experiments came also from an earlier study reporting that in schizophrenic patients, antibodies to a specific protein in neuroblastoma cells have been observed in patients with schizophrenia (Mazeh et al. 1998).

Due to the blood brain barrier, the brain is an immune privileged space, to which antibodies normally do not have access (Muldoon et al. 2013). Therefore, if antibodies specific to HPy or CJe should indeed be able to impair neuronal differentiation and synaptic signaling in vivo, the question remains: how could they pass the blood brain barrier in order to reach their targets in the developing and/or mature brain? At the moment, one can only speculate about this; however, it has already been shown that blood brain barrier permeability is increased in some schizophrenic patients and that this can be associated with increased IgG levels in the cerebrospinal fluid (Müller and Ackenheil 1995; Vasic et al. 2012). Also, during brain development, the blood brain barrier seems to be not yet fully established, suggesting that brain-reactive autoantibodies could indeed get direct access to neural precursor cells as well as young neurons and glial cells (Bauer et al. 2014).

In the end, results of the present study demonstrate also the limitations of an MPA analysis as it was performed here, since not for every protein, revealing an interaction with a given antibacterial antiserum on the MPA, a corresponding interaction partner by an alternative method like Western blot-analysis can be detected, and also the inverse case cannot be ruled out. This may be due to the fact that the proteins on the MPA were derived from cDNAs spotted on a PVDF membrane, which has then been translated in situ, using an in vitro translation system. Therefore these proteins lack most posttranslational modifications such as phosphorylation or glycosylation and therefore may be immunologically different from their natural occurring counterparts. Nevertheless it seems that the hexSelect MPA is a valuable tool for a first screening for possible immunological interaction partners of a given antiserum; however, every positive signal needs further confirmation and functional characterization in order to allow any statement on a putative pathological relevance!

In conclusion, results of the present study confirm and extend previous findings on interactions of antibacterial antibodies with human neuronal proteins, demonstrating for the first time cross-reactivity of antisera directed to the gastric mucosal bacterium *Helicobacter pylori* and the intestinal bacterium *Campylobacter jejuni* with the human synaptic protein *Syt5*, which seems to correlate also to functional changes in affected cells such as impaired ACh-dependent synaptic activity. These findings could be of importance for a better understanding of cellular and molecular mechanisms underlying the role of maternal bacterial infections for the increased schizophrenia risk in affected children later in life.

## Electronic Supplementary Material

Suppl. Fig. 1 Control experiment excluding unspecific interactions of the secondary antibodies with the protein spots on the hEXselect multiprotein array (MPA). As the red stained false color image of an X-ray film exposed to a hEXselect MPA incubated with secondary antibodies only (SAO) reveals, no pairwise immunoreactive spots are visible. (EPS 16552 kb) (PNG 2996 kb)

High Resolution (EPS 16552 kb)

Suppl. Fig. 2 Control incubation for the Western blot analysis of the HEK293 cells overexpression lysates for Syt5, Vglut1, Stmn4 and Ncan, with secondary antibodies only (SAO). Only a very weak background staining and almost no immunostained protein bands are visible. Likewise a control lysate of non transfected HEK293 cells, as well as an overexpression lysate of the non-reacting Srf protein is negative (PNG 330 kb)

High Resolution (EPS 336 kb)

Suppl. Fig. 3 Control incubations for the one- and two-dimensional Western blot analyses of SiMa human neuroblastoma cells with secondary antibodies only (SAO) and α-*HPy*. (a) Standard Western blot analysis of a whole cell protein extract of SiMa cells incubated with either α-*HPy* (blue), or SAO (red) revealing only a very weak background staining of cellular proteins by the secondary antibodies. The lack of unspecific interactions of the secondary antibodies is also confirmed by the corresponding overlay image. (b) Likewise the overlay image of a two dimensional Western blot analysis of a whole cell protein extract of SiMa-cells incubated with SAO (red) and α-*HPy* (blue) reveals also no specific staining by the secondary antibodies at all (PNG 1220 kb)

High Resolution (EPS 2594 kb)

Suppl. Fig. 4 Control incubations for the one- and two-dimensional Western blot analyses of SiMa human neuroblastoma cells with secondary antibodies only (SAO) and α-*CJe*. (a) Standard Western blot analysis of a whole cell protein extract of SiMa cells incubated with either α-*CJe* (blue), or SAO (red) revealing only a very weak background staining of cellular proteins by the secondary antibodies. The lack of unspecific interactions of the secondary antibodies is also confirmed by the corresponding overlay image. (b) Likewise the overlay image of a two dimensional Western blot analysis of a whole cell protein extract of SiMa-cells incubated with SAO (red) and α-*CJe* (blue) reveals also no specific staining by the secondary antibodies at all (PNG 1388 kb)

High Resolution (EPS 3438 kb)

Suppl. Fig. 5 Interactions of *α-HPy* and *α-CJe* with Syt5 in SiMa neuroblastoma cells, as revealed by a gene specific knockdown of Syt5 mRNA and protein due to the transfection with a commercial Syt5 shRNA expression vector. (a) Western blot analysis for Syt5 immunoreactivity in SiMa neuroblastoma cells transfected with the Syt5 shRNA expression vector, as compared to untreated cells, and also to cells transfected with a non-mammalian shRNA expression vector. (b) Western blot analysis for *α-HPy* immunoreactivity in SiMa neuroblastoma cells transfected with the Syt5 shRNA expression vector, as compared to untreated cells, and also to cells transfected with a nonmammalian shRNA expression vector. (c) Incubation of the same Western blot as shown in (a) and (b) with an antibody directed to β-actin confirms the amount of protein loaded on each lane to be identical. (d) Western blot analysis for Syt5 immunoreactivity in SiMa neuroblastoma cells transfected with the Syt5 shRNA expression vector, as compared to untreated cells, and also to cells transfected with a non-mammalian shRNA expression vector. (e) Western blot analysis for *α-HPy* immunoreactivity in SiMa neuroblastoma cells transfected with the Syt5 shRNA expression vector, as compared to untreated cells, and also to cells transfected with a non-mammalian shRNA expression vector. (f) Incubation of the same Western blot as shown in (d) and (e) with an antibody directed to β-actin confirms the amount of protein loaded on each lane to be identical. (PNG 1032 kb)

High Resolution (EPS 1159 kb)

## References

[CR1] Almamy A, Schwerk C, Schroten H, Ishikawa H, Asif AR, Reuss B (2017). Crossreactivity of an antiserum directed to the gram-negative bacterium Neisseria gonorrhoeae with the SNARE-complex protein Snap23 correlates to impaired exocytosis in SH-SY5Y cells. J Mol Neurosci.

[CR2] Babulas V, Factor-Litvak P, Goetz R, Schaefer CA, Brown AS (2006). Prenatal exposure to maternal genital and reproductive infections and adult schizophrenia. Am J Psychiatry.

[CR3] Bauer HC, Krizbai IA, Bauer H, Traweger A (2014). “You shall not pass”-tight junctions of the blood brain barrier. Front Neurosci.

[CR4] Berrios X, Quesney F, Morales A, Blazquez J, Bisno AL (1985). Are all recurrences of “pure” Sydenham chorea true recurrences of acute rheumatic fever?. J Pediatr.

[CR5] Bitanihirwe BK, Lim MP, Kelley JF, Kaneko T, Woo TU (2009). Glutamatergic deficits and parvalbumin-containing inhibitory neurons in the prefrontal cortex in schizophrenia. BMC Psychiatry.

[CR6] Bollag DM, Edelstein SJ (1994) Chapter 7: isoelectric focusing and two dimensional gel electrophoresis. In: Protein Methods. Wiley-Liss, Inc

[CR7] Büssow K, Cahill D, Nietfeld W, Bancroft D, Scherzinger E, Lehrach H, Walter G (1998). A method for global protein expression and antibody screening on high-density filters of an arrayed cDNA library. Nucleic Acids Res.

[CR8] Büssow K, Nordhoff E, Lübbert C, Lehrach H, Walter G (2000). A human cDNA library for high-throughput protein expression screening. Genomics.

[CR9] Caruso ML, Fucci L (1990). Histological identification of Helicobacter pylori in early and advanced gastric cancer. J Clin Gastroenterol.

[CR10] Chowdhury HH, Jevsek M, Kreft M, Mars T, Zorec R, Grubic Z (2005). Insulin-induced exocytosis in single, in vitro innervated human muscle fibres: a new approach. Pflugers Arch.

[CR11] Collins J (1957). Insulin resistance in schizophrenia. Med J Aust.

[CR12] Cunningham MW (2014). Rheumatic fever, autoimmunity, and molecular mimicry: the streptococcal connection. Int Rev Immunol.

[CR13] Dahm L, Klugmann F, Gonzalez-Algaba A, Reuss B (2010). Tamoxifen and raloxifene modulate gap junction coupling during early phases of retinoic acid-dependent neuronal differentiation of NTera2/D1 cells. Cell Biol Toxicol.

[CR14] de Hert M, Hautekeete M, De Wilde D, Peuskens J (1997). High prevalence of Helicobacter pylori in institutionalized schizophrenic patients. Schizophr Res.

[CR15] Dobbs RJ, Charlett A, Purkiss AG, Dobbs SM, Weller C, Peterson DW (1999). Association of circulating TNF-alpha and IL-6 with ageing and parkinsonism. Acta Neurol Scand.

[CR16] Eastwood SL, Harrison PJ (2005). Decreased expression of vesicular glutamate transporter 1 and complexin II mRNAs in schizophrenia: further evidence for a synaptic pathology affecting glutamate neurons. Schizophr Res.

[CR17] Edwards JL, Butler EK (2011). The pathobiology of *Neisseria gonorrhoeae* lower female genital tract infection. Front Microbiol.

[CR18] English JA, Dicker P, Föcking M, Dunn MJ, Cotter DR (2009). 2-D DIGE analysis implicates cytoskeletal abnormalities in psychiatric disease. Proteomics.

[CR19] Gaffield MA, Betz WJ (2006). Imaging synaptic vesicle exocytosis and endocytosis with FM dyes. Nat Protocols.

[CR20] Geppert M, Goda Y, Hammer RE, Li C, Rosahl TW, Stevens CF, Südhof TC (1994). Synaptotagmin I: a major Ca++-sensor for transmitter release at a central synapse. Cell.

[CR21] Giusti-Rodríguez P, Sullivan PF (2013). The genomics of schizophrenia: update and implications. J Clin Invest.

[CR22] Harland R, Antonova E, Owen GS, Broome M, Landau S, Deeley Q, Murray R (2009). A study of psychiatrists' concepts of mental illness. Psychol Med.

[CR23] Harrison PJ (1999). The neuropathology of schizophrenia. A critical review of the data and their interpretation. Brain.

[CR24] Henkel AW, Bieger SC (1994). Quantification of proteins dissolved in an electrophoresis sample buffer. Anal Biochem.

[CR25] Hoffman TA, Damus AJ, Sands L (1979). Evaluation of a gonococcal serologic test. Am J Clin Pathol.

[CR26] Hope S, Melle I, Aukrust P, Steen NE, Birkenaes AB, Lorentzen S, Agartz I, Ueland T, Andreassen OA (2009). Similar immune profile in bipolar disorder and schizophrenia: selective increase in soluble tumor necrosis factor receptor I and von Willebrand factor. Bipolar Disord.

[CR27] Horn S, Lueking A, Murphy D, Staudt A, Gutjahr C, Schulte K, König A, Landsberger M, Lehrach H, Felix SB, Cahill DJ (2006). Profiling humoral autoimmune repertoire of dilated cardiomyopathy (DCM) patients and development of a disease-associated protein chip. Proteomics.

[CR28] Hosák L, Silhan P, Hosáková J (2012). Genome-wide association studies in schizophrenia, and potential etiological and functional implications of their results. Acta Med (Hradec Kralove).

[CR29] Iezzi M, Kouri G, Fukuda M, Wollheim CB (2004). Synaptotagmin V and IX isoforms control Ca2+-dependent insulin exocytosis. J Cell Sci.

[CR30] Iorio R, Lennon VA (2012). Neural antigen-specific autoimmune disorders. Immuological Rev.

[CR31] Kahn RS, Sommer IE (2015). The neurobiology and treatment of first-episode schizophrenia. Mol Psychiatry.

[CR32] Khandaker GM, Zimbron J, Lewis G, Jones PB (2013). Prenatal maternal infection, neurodevelopment and adult schizophrenia: a systematic review of population-based studies. Psychol Med.

[CR33] Kirvan CA, Swedo SE, Kurahara D, Cunningham MW (2006). Streptococcal mimicry and antibody-mediated cell signaling in the pathogenesis of Sydenham's chorea. Autoimmunity.

[CR34] Kontkanen O, Törönen P, Lakso M, Wong G, Castrén E (2002). Antipsychotic drug treatment induces differential gene expression in the rat cortex. J Neurochem.

[CR35] Kountouras J, Deretzi G, Zavos C, Karatzoglou P, Touloumis L, Nicolaides T, Chatzopoulos D, Venizelos I (2005). Association between Helicobacter pylori infection and acute inflammatory demyelinating polyradiculoneuropathy. Eur J Neurol.

[CR36] Kountouras J, Boziki M, Gavalas E, Zavos C, Deretzi G, Grigoriadis N, Tsolaki M, Chatzopoulos D, Katsinelos P, Tzilves D, Zabouri A, Michailidou I (2009). Increased cerebrospinal fluid Helicobacter pylori antibody in Alzheimer’s disease. Int J Neurosci.

[CR37] Labenz J, Börsch G (1994). Evidence for the essential role of Helicobacter pylori in gastric ulcer disease. Gut.

[CR38] Laemmli UK (1970). Cleavage of structural proteins during the assembly of the head of bacteriophage T4. Nature.

[CR39] Lee YH, Cherkerzian S, Seidman LJ, Papandonatos GD, Savitz DA, Tsuang MT, Goldstein JM, Buka SL (2019) Maternal bacterial infection during pregnancy and offspring risk of psychotic disorders: variation by severity of infection and offspring sex. Am J Psychiatry [Epub ahead of print] 10.1176/appi.ajp.2019.18101206 PMC6939139 31581799

[CR40] Lenzi C, Stevens J, Osborn D, Hannah MJ, Bierings R, Carter T (2019) Synaptotagmin 5 regulates Ca2+−dependent Weibel–Palade body exocytosis in human endothelial cells. Journal of cell science 132, jcs221952 10.1242/jcs.221952 PMC6565431 30659119

[CR41] Levitz SM, Diamond RD (1985). A rapid colorimetric assay of fungal viability with the tetrazolium salt MTT. J Infect Dis.

[CR42] Li W, Minohara M, Su JJ, Matsuoka T, Osoegawa M, Ishizu T, Kira J (2007). Helicobacter pylori infection is a potential protective factor against conventional multiple sclerosis in the Japanese population. Neuroimmunol.

[CR43] Lindenmayer JP, Nathan AM, Smith RC (2001). Hyperglycemia associated with the use of atypical antipsychotics. J Clin Psychiatry.

[CR44] Marini P, RA ML, Treuner C, Bruchelt G, Böhm W, Wolburg H, Schweizer P, Girgert R (1999). SiMa, a new neuroblastoma cell line combining poor prognostic cytogenetic markers with high adrenergic differentiation. Cancer Genet Cytogenet.

[CR45] Marshall BJ, Warren JR (1983). Unidentified curved bacilli on gastric epithelium in active chronic gastritis. Lancet.

[CR46] Martins-de-Souza D, Maccarrone G, Wobrock T, Zerr I, Gormanns P, Reckow S, Falkai P, Schmitt A, Turck CW (2010). Proteome analysis of the thalamus and cerebrospinal fluid reveals glycolysis dysfunction and potential biomarkers candidates for schizophrenia. J Psychiatr Res.

[CR47] Masopust J, Malý R, Andrýs C, Vališ M, Bažant J, Hosák L (2011). Markers of thrombogenesis are activated in unmedicated patients with acute psychosis: a matched case control study. BMC Psychiatry.

[CR48] Maycox PR, Kelly F, Taylor A, Bates S, Reid J, Logendra R, Barnes MR, Larminie C, Jones N, Lennon M, Davies C, Hagan JJ, Scorer CA, Angelinetta C, Akbar T, Hirsch S, Mortimer AM, Barnes TRE, de Belleroche J (2009). Analysis of gene expression in two large schizophrenia cohorts identifies multiple changes associated with nerve terminal function. Mol Psychiatry.

[CR49] Mazeh D, Sirota P, Patya M, Novogrodsky A (1998). Antibodies to neuroblastoma cell line proteins in patients with schizophrenia. J Neuroimmunol.

[CR50] Melkersson KI, Hulting AL, Brismar KE (1999). Different influences of classical antipsychotics and clozapine on glucose-insulin homeostasis in patients with schizophrenia or related psychoses. J Clin Psychiatry.

[CR51] Mikoshiba K, Fukuda M, Moreira JE, Lewis FMT, Sugimori M, Niinobe M, Llinas R (1995). Role of the C2A domain of synaptotagmin in transmitter release as determined by specific antibody injection into the squid giant synapse preterminal. Proc Natl Acad Sci U S A.

[CR52] Montecucco C, Rappuoli R (2001). Living dangerously: how Helicobacter pylori survives in the human stomach. Nat Rev Mol Cell Biol.

[CR53] Moran AP, Prendergast MM (2001). Molecular mimicry in Campylobacter jejuni and Helicobacter pylori lipopolysaccharides: contribution of gastrointestinal infections to autoimmunity. J Autoimmun.

[CR54] Muldoon LL, Alvarez JI, Begley DJ, Boado RJ, del Zoppo GJ, Doolittle ND, Engelhardt B, Hallenbeck JM, Lonser RR, Ohlfest JR, Prat A, Scarpa M, Smeyne RJ, Drewes LR, Neuwelt EA (2013). Immunologic privilege in the central nervous system and the blood–brain barrier. J Cereb Blood Flow Metab.

[CR55] Müller N, Ackenheil M (1995). Immunoglobulin and albumin content of cerebrospinal fluid in schizophrenic patients: relationship to negative symptomatology. Schizophr Res.

[CR56] Murray RM, O'Callaghan E, Castle DJ, Lewis SW (1992). A neurodevelopmental approach to the classification of schizophrenia. Schizophr Bull.

[CR57] Murray RM, Bhavsar V, Tripoli G, Howes O (2017). 30 years on: how the neurodevelopmental hypothesis of schizophrenia morphed into the developmental risk factor model of psychosis. Schizophr Bull.

[CR58] Noll R (2004). Historical review: autointoxication and focal infection theories of dementia praecox. World J Biol Psychiatry.

[CR59] Noll R (2007). Kraepelin’s ‘lost biological psychiatry’? Autointoxication, organotherapy and surgery for dementia praecox. Hist Psychiatry.

[CR60] Oates SA, Falkler WA, Joseph JM, Warfel LE (1977). Asymptomatic females: detection of antibody activity to gonococcal pili antigen by radioimmunoassay. J Clin Microbiol.

[CR61] Oldstone MB (1998). Molecular mimicry and immune-mediated diseases. FASEB J.

[CR62] Reuss B, Asif AR (2014). Antibodies directed to the gram-negative bacterium *Neisseria gonorrhoeae* cross-react with the 60 kDa heat shock protein and lead to impaired neurite outgrowth in NTera2/D1 cells. J Mol Neurosci.

[CR63] Reuss B, Asif AR, Almamy A, Schwerk C, Schroten H, Ishikawa H, Drummer C, Behr R (2016). Antisera against Neisseria gonorrhoeae cross-react with specific brain proteins of the common marmoset monkey and other nonhuman primate species. Brain Res.

[CR64] Roubaud-Baudron C, Krolak-Salmon P, Quadrio I, Mégraud F, Salles N (2012). Impact of chronic Helicobacter pylori infection on Alzheimer’s disease: preliminary results. Neurobiol Aging.

[CR65] Schimmelbusch WH, Mueller PS, Sheps J (1971). The positive correlation between insulin resistance and duration of hospitalization in untreated schizophrenia. Br J Psychiatry.

[CR66] Schultz CC, Mühleisen TW, Nenadic I, Koch K, Wagner G, Schachtzabel C, Siedek F, Nöthen MM, Rietschel M, Deufel T, Kiehntopf M, Cichon S, Reichenbach JR, Sauer H, Schlösser RG (2014). Common variation in NCAN, a risk factor for bipolar disorder and schizophrenia, influences local cortical folding in schizophrenia. Psychol Med.

[CR67] Simeone JC, Ward AJ, Rotella P, Collins J, Windisch R (2015). An evaluation of variation in published estimates of schizophrenia prevalence from 1990─2013: a systematic literature review. BMC Psychiatry.

[CR68] Sørensen HJ, Mortensen EL, Reinisch JM, Mednick SA (2009). Association between prenatal exposure to bacterial infection and risk of schizophrenia. Schizophrenia Bull.

[CR69] Speed B, Kaldor J, Cavanagh P (1984). Guillain-Barré syndrome associated with Campylobacter jejuni enteritis. J Inf Secur.

[CR70] Südhof TC (1995). The synaptic vesicle cycle: a cascade of protein–protein interactions. Nature.

[CR71] Tandon R, Nasrallah HA, Keshavan MS (2009). Schizophrenia, “just the facts” 4. Clinical features and conceptualization. Schizophr Res.

[CR72] Tret'iakov A, Karpov AG, Polushin PI, Zakharchenko SP (2006). Gastric ulcer accompanying schizophrenia: variants of combination and particulars of development. Eksp Klin Gastroenterol.

[CR73] Vasic N, Connemann BJ, Wolf RC, Tumani H, Brettschneider J (2012). Cerebrospinal fluid biomarker candidates of schizophrenia: where do we stand?. Eur Arch Psychiatry Clin Neurosci.

[CR74] Verma SK, Subramaniam M, Liew A, Poon LY (2009). Metabolic risk factors in drug-naive patients with first-episode psychosis. J Clin Psychiatry.

[CR75] Wang KS, Liu XF, Aragam N (2010). A genome-wide meta-analysis identifies novel loci associated with schizophrenia and bipolar disorder. Schizophr Res.

[CR76] Wang P, Cai J, Ni J, Zhang J, Tang W, Zhang C (2016). The NCAN gene: schizophrenia susceptibility and cognitive dysfunction. Neuropsychiatr Dis Treat.

[CR77] Wijdicks EF, Klein CJ (2017). Guillain-Barré syndrome. Mayo Clin Proc.

[CR78] Yilmaz Y, Gul CB, Arabul M, Eren MA (2008). Helicobacter pylori: a role in schizophrenia?. Med Sci Monit.

