# Supplementary figures and images for: Interactions of Antibodies to the Gram-Negative Gastric Bacterium Helicobacter pylori with the Synaptic Calcium Sensor Synaptotagmin 5, Correlate to Impaired Vesicle Recycling in SiMa Human Neuroblastoma Cells
Source: J Mol Neurosci. 2020 Aug 28;71(3):481–505. doi: 10.1007/s12031-020-01670-0 (PMC7851109; doi:10.1007/s12031-020-01670-0)

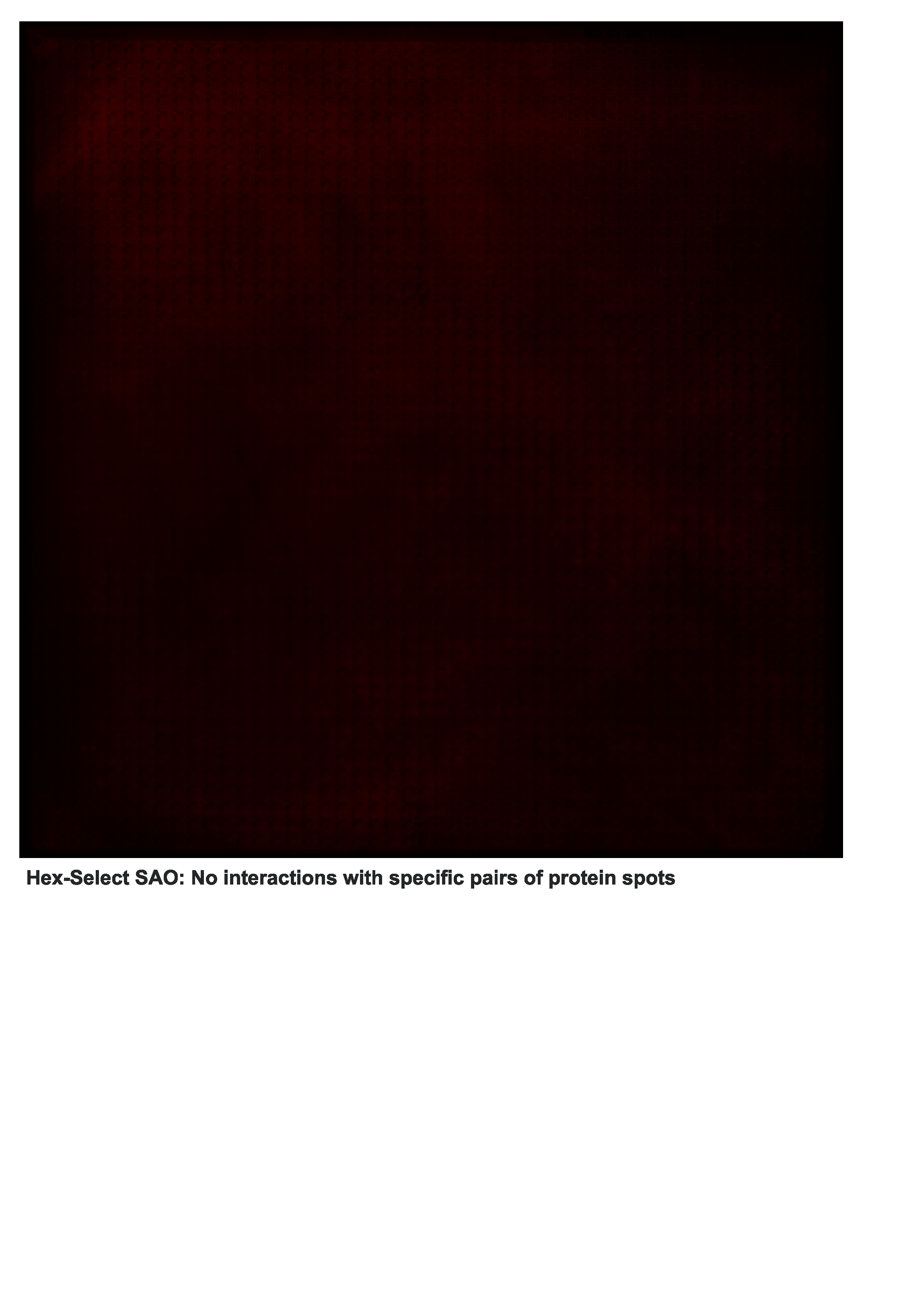

Supplement: Supplementary file 1 — Control experiment excluding unspecific interactions of the secondary antibodies with the protein spots on the hEXselect multiprotein array (MPA). As the red stained false color image of an X-ray film exposed to a hEXselect MPA incubated with secondary antibodies only (SAO) reveals, no pairwise immunoreactive spots are visible. (EPS 16552 kb) (PNG 2996 kb) [file 12031_2020_1670_Fig11_ESM.png]

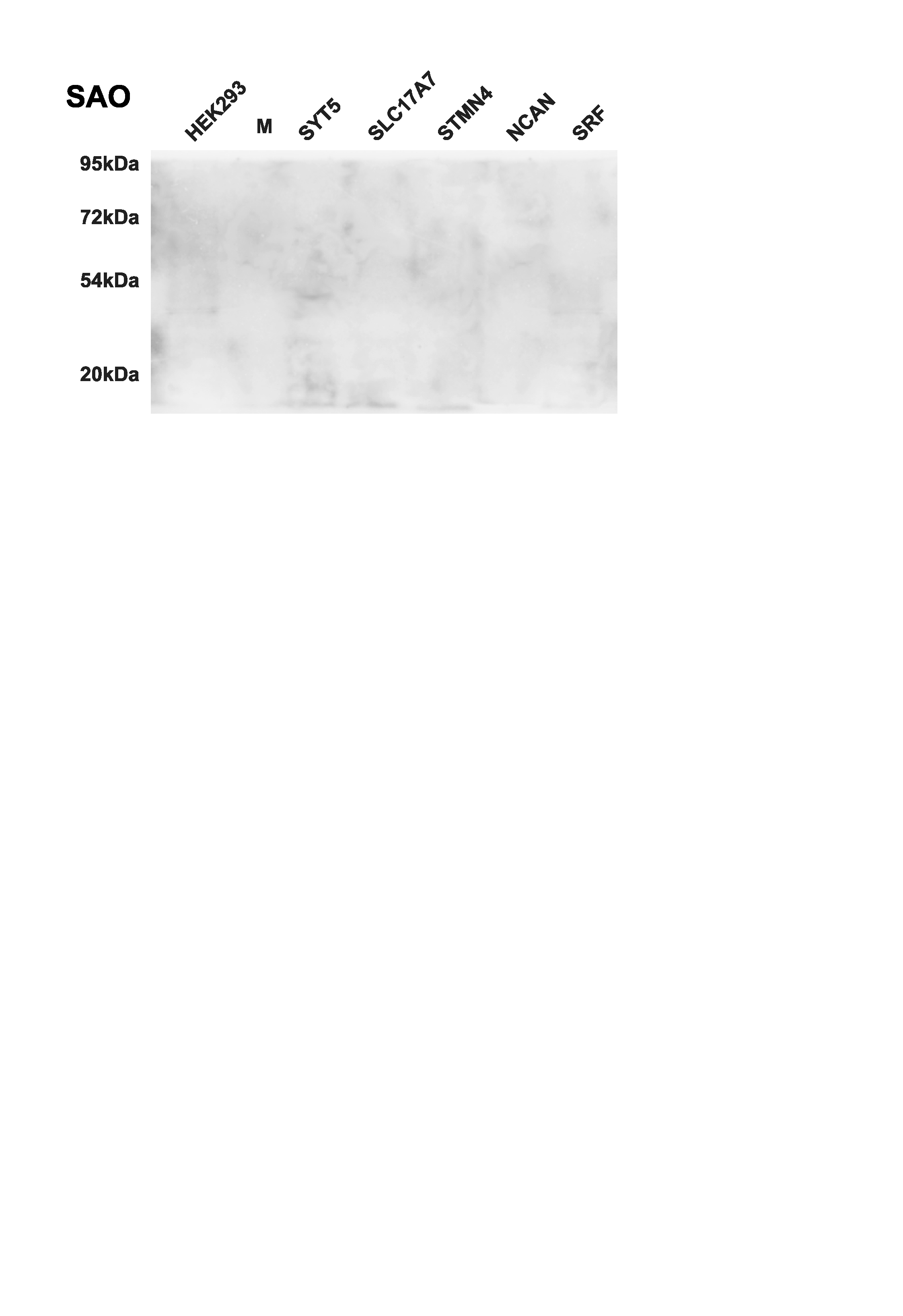

Supplement: Supplementary file 3 — Control incubation for the Western blot analysis of the HEK293 cells overexpression lysates for Syt5, Vglut1, Stmn4 and Ncan, with secondary antibodies only (SAO). Only a very weak background staining and almost no immunostained protein bands are visible. Likewise a control lysate of non transfected HEK293 cells, as well as an overexpression lysate of the non-reacting Srf protein is negative (PNG 330 kb) [file 12031_2020_1670_Fig12_ESM.png]

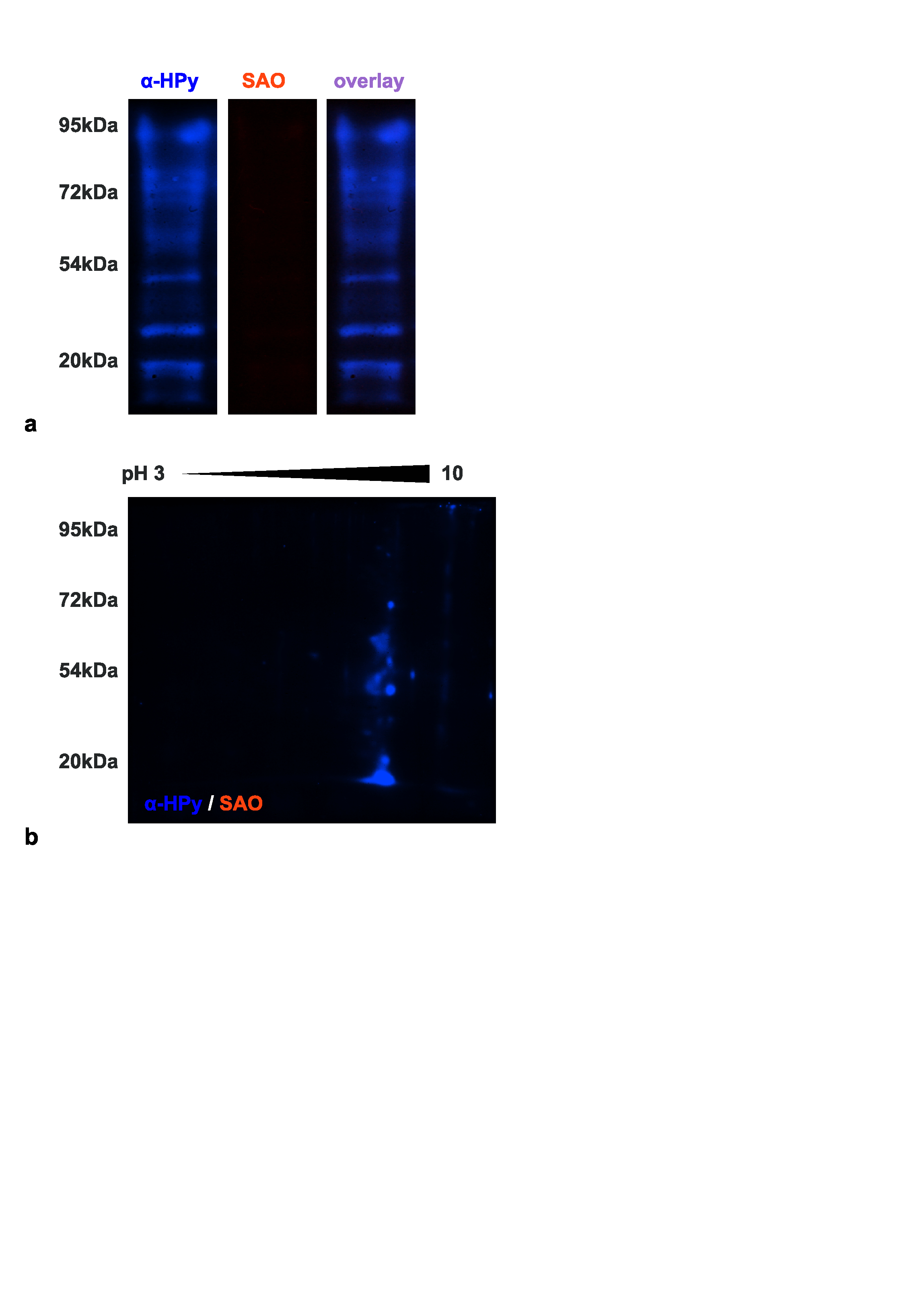

Supplement: Supplementary file 5 — Control incubations for the one- and two-dimensional Western blot analyses of SiMa human neuroblastoma cells with secondary antibodies only (SAO) and α-HPy. (a) Standard Western blot analysis of a whole cell protein extract of SiMa cells incubated with either α-HPy (blue), or SAO (red) revealing only a very weak background staining of cellular proteins by the secondary antibodies. The lack of unspecific interactions of the secondary antibodies is also confirmed by the corresponding overlay image. (b) Likewise the overlay image of a two dimensional Western blot analysis of a whole cell protein extract of SiMa-cells incubated with SAO (red) and α-HPy (blue) reveals also no specific staining by the secondary antibodies at all (PNG 1220 kb) [file 12031_2020_1670_Fig13_ESM.png]

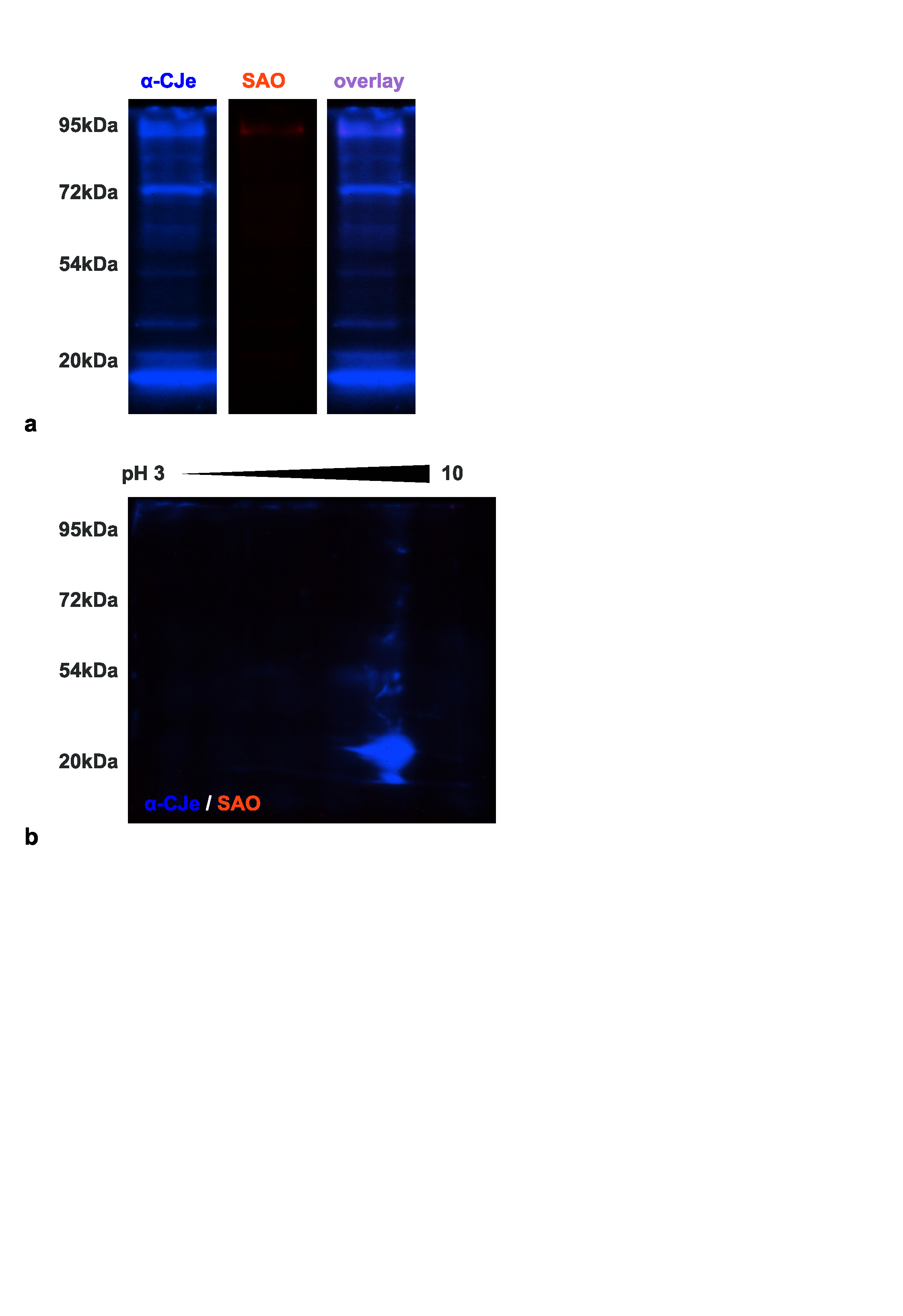

Supplement: Supplementary file 7 — Control incubations for the one- and two-dimensional Western blot analyses of SiMa human neuroblastoma cells with secondary antibodies only (SAO) and α-CJe. (a) Standard Western blot analysis of a whole cell protein extract of SiMa cells incubated with either α-CJe (blue), or SAO (red) revealing only a very weak background staining of cellular proteins by the secondary antibodies. The lack of unspecific interactions of the secondary antibodies is also confirmed by the corresponding overlay image. (b) Likewise the overlay image of a two dimensional Western blot analysis of a whole cell protein extract of SiMa-cells incubated with SAO (red) and α-CJe (blue) reveals also no specific staining by the secondary antibodies at all (PNG 1388 kb) [file 12031_2020_1670_Fig14_ESM.png]

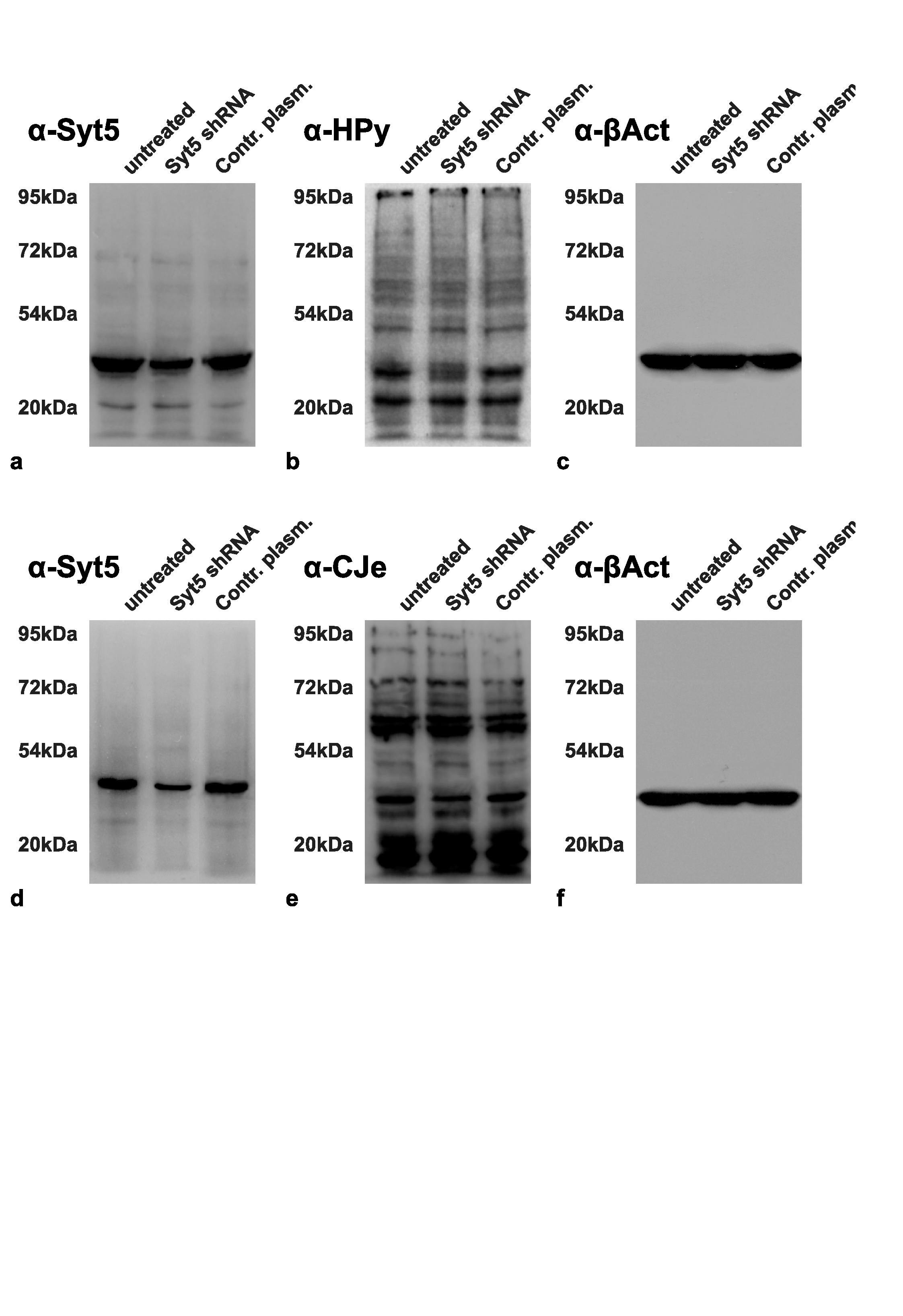

Supplement: Supplementary file 9 — Interactions of α-HPy and α-CJe with Syt5 in SiMa neuroblastoma cells, as revealed by a gene specific knockdown of Syt5 mRNA and protein due to the transfection with a commercial Syt5 shRNA expression vector. (a) Western blot analysis for Syt5 immunoreactivity in SiMa neuroblastoma cells transfected with the Syt5 shRNA expression vector, as compared to untreated cells, and also to cells transfected with a non-mammalian shRNA expression vector. (b) Western blot analysis for α-HPy immunoreactivity in SiMa neuroblastoma cells transfected with the Syt5 shRNA expression vector, as compared to untreated cells, and also to cells transfected with a nonmammalian shRNA expression vector. (c) Incubation of the same Western blot as shown in (a) and (b) with an antibody directed to β-actin confirms the amount of protein loaded on each lane to be identical. (d) Western blot analysis for Syt5 immunoreactivity in SiMa neuroblastoma cells transfected with the Syt5 shRNA expression vector, as compared to untreated cells, and also to cells transfected with a non-mammalian shRNA expression vector. (e) Western blot analysis for α-HPy immunoreactivity in SiMa neuroblastoma cells transfected with the Syt5 shRNA expression vector, as compared to untreated cells, and also to cells transfected with a non-mammalian shRNA expression vector. (f) Incubation of the same Western blot as shown in (d) and (e) with an antibody directed to β-actin confirms the amount of protein loaded on each lane to be identical. (PNG 1032 kb) [file 12031_2020_1670_Fig15_ESM.png]
